# Mitigating the risk of antimalarial resistance via covalent dual-subunit inhibition of the *Plasmodium* proteasome

**DOI:** 10.1016/j.chembiol.2023.03.002

**Published:** 2023-03-22

**Authors:** Ioanna Deni, Barbara H. Stokes, Kurt E. Ward, Kate J. Fairhurst, Charisse Flerida A. Pasaje, Tomas Yeo, Shirin Akbar, Heekuk Park, Ryan Muir, Daniella S. Bick, Wenhu Zhan, Hao Zhang, Yi Jing Liu, Caroline L. Ng, Laura A. Kirkman, Jehad Almaliti, Alexandra E. Gould, Maëlle Duffey, Anthony J. O’Donoghue, Anne-Catrin Uhlemann, Jacquin C. Niles, Paula C.A. da Fonseca, William H. Gerwick, Gang Lin, Matthew Bogyo, David A. Fidock

**Affiliations:** 1 Center for Malaria Therapeutics and Antimicrobial Resistance and Department of Microbiology and Immunology, Columbia University Irving Medical Center, New York, NY, USA; 2 Department of Biological Engineering, Massachusetts Institute of Technology, Cambridge, MA, USA; 3 School of Molecular Biosciences, University of Glasgow, Glasgow, Scotland, UK; 4 Division of Infectious Diseases, Department of Medicine, Columbia University Irving Medical Center, New York, NY, USA; 5 Department of Pathology, Stanford University School of Medicine, Stanford, CA, USA; 6 Department of Microbiology and Immunology, Weill Cornell Medicine, New York, NY, USA; 7 Global Center for Health Security, University of Nebraska Medical Center, Omaha, NE, USA; 8 Department of Biology, University of Nebraska Omaha, Omaha, NE, USA; 9 Department of Pathology and Microbiology, University of Nebraska Medical Center, Omaha, NE, USA; 10 Division of Infectious Diseases, Department of Medicine, Weill Cornell Medicine, New York, NY; 11 Scripps Institution of Oceanography, University of California San Diego, La Jolla, CA, USA; 12 Department of Pharmaceutical Sciences, College of Pharmacy, The University of Jordan, Amman, Jordan; 13 Takeda Development Center Americas, Inc., Cambridge, MA 02139, USA; 14 Medicines for Malaria Venture, Geneva, Switzerland; 15 Skaggs School of Pharmacy and Pharmaceutical Sciences, University of California San Diego, La Jolla, CA, USA; 16 Present address: INSERM U1016, Centre National de la Recherche Scientifique (CNRS), Unité Mixte de Recherche (UMR) 8104, Université de Paris, Institut Cochin, Paris, France; 17 Present address: Wellcome Center for Integrative Parasitology, School of Infection & Immunity, University of Glasgow, Glasgow, Scotland, UK; 18 Present address: iCarbonX (Shenzhen) Co., Ltd., Shenzhen, China; 19 Present address: Global Antibiotic Research and Development Partnership, Geneva, Switzerland; 20 These authors contributed equally; 21 Lead contact

## Abstract

The *Plasmodium falciparum* proteasome constitutes a promising antimalarial target, with multiple chemotypes potently and selectively inhibiting parasite proliferation and synergizing with the first-line artemisinin drugs, including against artemisinin-resistant parasites. We compared resistance profiles of vinyl sulfone, epoxyketone, macrocyclic peptide, and asparagine ethylenediamine inhibitors and report that the vinyl sulfones were potent even against mutant parasites resistant to other proteasome inhibitors and did not readily select for resistance, particularly WLL that displays covalent and irreversible binding to the catalytic β2 and β5 proteasome subunits. We also observed instances of collateral hypersensitivity, whereby resistance to one inhibitor could sensitize parasites to distinct chemotypes. Proteasome selectivity was confirmed using CRISPR/Cas9-edited mutant and conditional knockdown parasites. Molecular modeling of proteasome mutations suggested spatial contraction of the β5 P1 binding pocket, compromising compound binding. Dual targeting of *P. falciparum* proteasome subunits using covalent inhibitors provides a potential strategy for restoring artemisinin activity and combating the spread of drug-resistant malaria.

## Graphical abstract

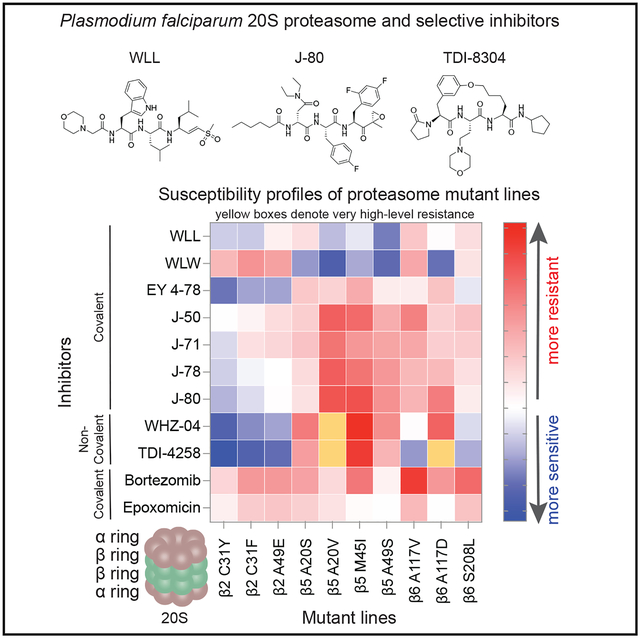

## In brief

Inhibitors of the *Plasmodium falciparum* proteasome have emerged as leading antimalarial candidates. Herein, Deni and Stokes et al. profile a range of diverse chemotypes and identify the vinyl sulfone WLL as the least susceptible to resistance, likely attributable to its covalent, irreversible binding to the β2 and β5 catalytic subunits.

## INTRODUCTION

*Plasmodium falciparum* malaria is a leading cause of mortality among young children in sub-Saharan Africa, who comprised the vast majority of the estimated 619,000 deaths globally in 2021.^1^ Recent reports of clinically confirmed *de novo* emergence of *P. falciparum* partial resistance to first-line artemisinin (ART) derivatives in East Africa, following the spread of ART partial resistance throughout Southeast Asia, portend a potential worsening of malaria’s impact across the African continent.^2–6^ To counter drug-resistant malaria, a particularly promising approach is selectively targeting the *P. falciparum* proteasome.^7^

The proteasome is a multi-subunit proteolytic enzyme complex that plays an integral role in maintaining cellular homeostasis in all eukaryotic organisms, including *Plasmodium* spp. The proteasome contains a 20S core particle with catalytic activity mediated by the β1, β2, and β5 subunits. Access to this core is predominantly regulated by the coupled 19S regulatory particle.^8^ This protein complex controls the removal of proteins specifically tagged by polyubiquitin, including ones that are damaged or that temporally regulate diverse processes, including cell cycle progression. Recent evidence also suggests that the *P. falciparum* 20S proteasome might be secreted into extracellular vesicles that modulate the mechanical properties of native human red blood cells (RBCs) by remodeling their cytoskeletal network, thereby priming RBCs for parasite invasion.^9^ Proteasome inhibitors block the development of multiple stages of the *Plasmodium* life cycle, including oocysts and sporozoites, and broadly interfere with progression through the liver and blood stages, including gametocytogenesis.^10–13^ Novartis, GlaxoSmithKline, and the University of Dundee have realized the value of targeting parasite proteasomes and have developed clinical candidates that inhibit the activity of kinetoplastid proteasomes.^1,14–18^ These compounds are being developed as therapeutics to treat human African trypanosomiasis, Chagas disease, and leishmaniasis.

Large-scale screening and structure-guided chemical optimization efforts have recently identified highly selective inhibitors of *Plasmodium* spp. proteasomes.^13,19–27^ Further refinements to inhibitor design have been made possible by the elucidation of cryoelectron microscopy (cryo-EM) structures of the *P. falciparum* 20S proteasome.^21,28^ These compounds fall broadly into two classes: covalent inhibitors that form irreversible or slowly reversible bonds with the catalytic threonine in the active sites of the catalytic β subunits, or non-covalent inhibitors that reversibly bind the proteasome to block its proteolytic activity. For covalent inhibitors, potency is based on both the initial binding interactions and the subsequent rate of chemical bond formation. Thus, inhibition is time-dependent, and extended exposure to a compound can help compensate for reduced binding affinity to mutant proteasomes. Of the reported covalent inhibitors, the irreversible vinyl sulfones were previously found to have a minimal risk of resistance *in vitro*.^21,29^ This is a key feature of these inhibitors, given that several advanced antimalarial candidates have selected for moderate to highly resistant parasites in human clinical trials.^30–32^ For those candidates, high-grade resistance *in vitro* was correlated with *in vivo* recrudescence, highlighting the importance of understanding resistance liabilities prior to initiating clinical development.^33–35^

Several *Plasmodium*-selective proteasome inhibitors have been shown to synergize with ART *in vitro*, presumably because these compounds interfere with the parasite’s response to cellular damage induced by ART treatment.^21,23,29,36–38^ ART acts by forming reactive radical species that can alkylate a broad array of parasite biomolecules, causing proteotoxic stress among other forms of cellular damage, such as impaired redox homeostasis.^39,40^ Importantly, mutations in the parasite protein K13 (PfKelch13), which mediate resistance to ART, do not interfere with synergy between proteasome inhibitors and ART, including *in vitro* with *P. falciparum* cultured asexual blood-stage parasites and *in vivo* in *Plasmodium berghei*-infected mice.^27,29,37^ This synergy reinforces the appeal of developing *Plasmodium*-selective proteasome inhibitors as potential new antimalarial medicines.^8,41^

Here we examine a panel of inhibitors that represent the main classes of compounds that are selective for *Plasmodium* proteasomes. Our study, which includes drug susceptibility assays with mutant parasites, *in vitro* resistance selections, reverse genetics, and molecular modeling, sheds light on compound specificity and identifies classes less likely to readily succumb to resistance, with dual covalent inhibition of the β2 and β5 active sites appearing particularly favorable. These data will help to inform future drug development efforts targeting the *P. falciparum* proteasome.

## RESULTS

### Mutations in the *P. falciparum* 26S proteasome confer distinct patterns of resistance to different inhibitor classes

**Figure 1.**
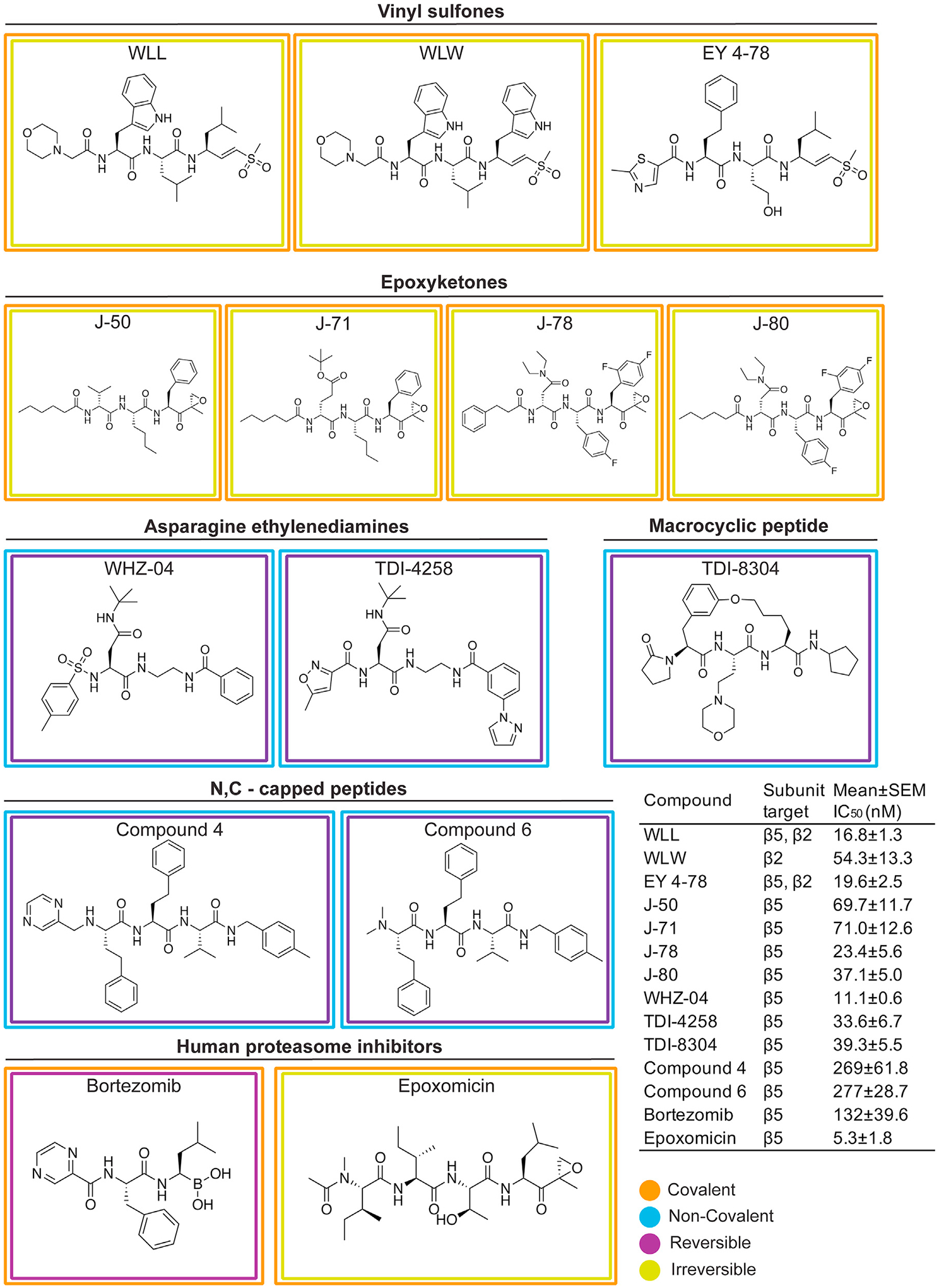
Structures of proteasome inhibitors employed in this study Chemical structures and binding properties of *Plasmodium*-selective and non-selective proteasome inhibitors tested herein. Mean IC_50_ ± SEM for inhibitors tested against Dd2 parasites is shown (N = 2–20, n = 2; see Table S1). Binding properties (covalent or not, reversible or not) for the *Plasmodium*-selective inhibitors are described in the following citations: vinyl sulfones,^21,23,43^ epoxyketones,^44,45^ asparagine ethylenediamines (AsnEDAs),^13,24^ the macrocyclic peptide TDI-8304,^27^ and the N,C-capped peptides listed as compounds 4 and 6.^20^ The binding properties and potent antiplasmodial activity of the human proteasome inhibitors epoxomicin and bortezomib have also been previously described.^46–49^

Compound screens and structure- and function-based inhibitor design have yielded compounds with diverse chemotypes that selectively inhibit the *P. falciparum* proteasome.^13,21,22,42^ Resistance to specific chemotypes can be mediated by point mutations that reside mostly within or at the interfaces of the catalytic β subunits that comprise the main substrate-binding pockets of the parasite proteasome.^13,25,29^ To determine the degree to which these mutations mediate resistance to different classes of inhibitors, we profiled chemically diverse compounds (Figure 1) against a panel of proteasome mutant and wild-type (WT) parasite lines in 72 h dose-response assays. These mutant lines harbor single-nucleotide polymorphisms (SNPs) in the β subunits of the Pf20S proteasome core particle or in the Pf19S proteasome regulatory particle, and were previously generated from *in vitro* resistance selection studies (Table 1). The mutant lines were selected from the Southeast Asian lines Dd2 (clone B2), Cam3.II (K13 WT or C580Y mutant), or V1/S (K13 WT or C580Y mutant). Compounds were chosen to include several different classes of proteasome inhibitors with different modes of action, which can be classified as covalent irreversible (vinyl sulfones and epoxyketones), covalent reversible (bortezomib, a boronate), or non-covalent reversible (with no reactive electrophile, represented by two asparagine ethylenediamines [AsnEDAs], the macrocyclic peptide TDI-8304, and two N,C-capped peptides). They also have different proteasome subunit selectivity patterns, with most predominantly inhibiting either β5 or β2, while some vinyl sulfones target both these subunits (Figure 1). From these dose-response assays, we determined the half-maximal growth inhibitory concentration (IC_50_) of each compound against asynchronous asexual blood-stage *P. falciparum* cultures (Figure 1, inset). Bortezomib and epoxomicin are non-selective inhibitors that also bind human proteasomes in addition to their antiplasmodial activity and were included for comparative purposes.^11,13,42^

**Figure 2.**
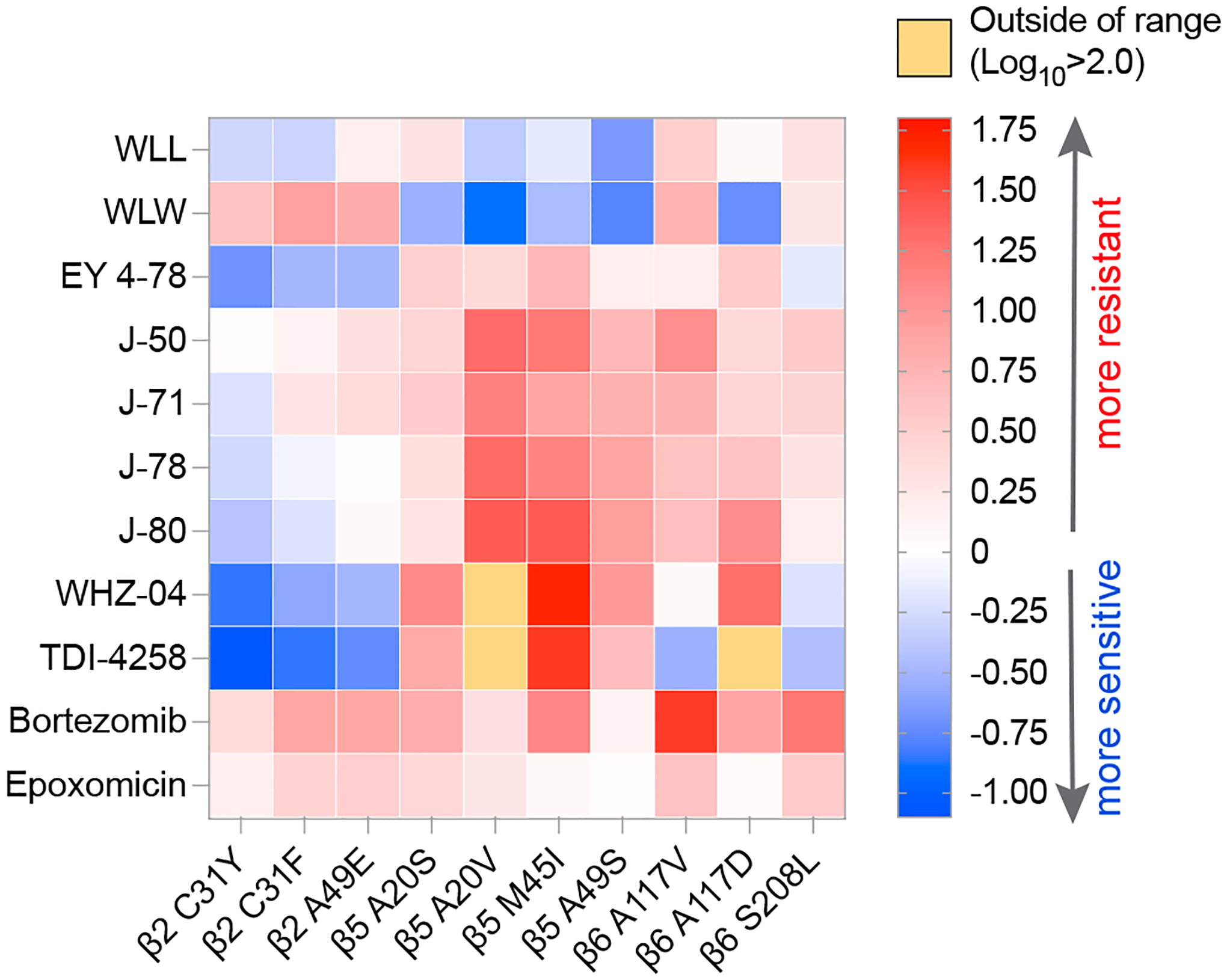
Heatmap of altered susceptibility profiles of a panel of *P. falciparum* proteasome mutant lines tested against a diverse panel of proteasome inhibitors IC_50_ values were obtained against each mutant line and its corresponding parental control for each of the listed compounds. Ratios of the IC_50_ value against the mutant line divided by the IC_50_ against the parental control line were calculated as the IC_50_ fold change and log_10_ transformed, and are presented as a heatmap to visualize instances of resistance or collateral sensitivity to various chemotypes for the individual mutant lines. The scale bar represents the log_10_-fold change. Mean ± SEM IC_50_ values for WT and mutant lines and statistical analyses are presented in Figure S1 and Table S1. Untransformed fold changes are listed in Table S2.

Results for dose-response assays testing the activity of the selected inhibitors against our panel of proteasome WT and mutant lines are represented as a heatmap in Figure 2, which shows the log_10_-fold change (the ratio of the IC_50_ of the compound tested against a mutant line divided by its IC_50_ against the corresponding WT parental line) for each compound against each mutant line. Mean ± SEM IC_50_ values are shown in Figure S1 as bar charts that include asterisks to indicate statistically significant changes, with numerical values and fold shifts provided in Tables S1 and S2, respectively.

We first tested the three vinyl sulfone inhibitors WLL, WLW, and EY 4–78, which are all covalent, irreversible inhibitors of the *Plasmodium* proteasome.^21,23^ WLL and EY 4–78 inhibit both the Pf20S β5 and β2 subunits, whereas WLW primarily inhibits β2^21^ (Figure 1). WLL and WLW exhibited relatively small shifts in their IC_50_ values when tested against our proteasome mutant lines compared with WT parental controls (Figure 2 and Table S2). For WLL, we observed 2- to 3-fold higher IC_50_ values against the WLL-selected β5 A20S and β6 A117V mutant lines^29^ compared with their respective parental lines. Collateral sensitivity was also observed, most prominently in β5 A49S mutant parasites (selected with TDI-4258; Table 1) that yielded 5-fold lower IC_50_ values relative to the corresponding WT parasites (Figure 2). Interestingly, parasites harboring an A20V mutation at the same residue as the WLL-selected A20S mutation became more sensitive to WLL as well. The β5 A20V mutant line was selected using the boronate compound MMV1579506, a covalent reversible inhibitor from Takeda that we previously profiled for resistance (Figures S2A and S2B). These data suggest that resistance to proteasome inhibitors is highly compound-specific, and that mutations selected with one compound can lead to collateral sensitivity to other inhibitor classes.

For WLW, we observed 4- to 8-fold higher IC_50_ values against the WLW-selected β2 mutant lines. Interestingly, WLW showed increased potency (with up to an 8-fold lower IC_50_) against all lines with mutations in β5, as well as against the β6 A117D mutant line, compared with their respective parental lines (Figures 2 and S1; Tables S1 and S2). Nonetheless, some cross resistance between WLL and WLW was observed, with WLW exhibiting somewhat reduced activity against the WLL-selected β6 A117V and β6 S208L mutant lines. These results imply that, in the case of vinyl sulfones, compounds within the same class can have significantly different resistance profiles depending on their subunit specificities.

Optimization of these first-generation *Plasmodium*-specific vinyl sulfones resulted in generation of a new lead molecule, EY 4–78 (previously “inhibitor 28”), with less cross-reactivity toward the human proteasome as well as improved solubility and oral bioavailability.^23^ Like WLL, EY 4–78 also inhibits both the β2 and β5 20S subunits. We tested EY 4–78 (Figure 1) against our panel of mutant parasite lines, including WLL- and WLW-selected mutants. Despite chemical similarities between EY 4–78 and WLL, certain mutations conferred distinct patterns of resistance to the two compounds. For example, the β5 M45I mutation, selected with MPI-12,^25^ conferred up to a 5-fold gain of resistance to EY 4–78, but resulted in sensitization of parasites to WLL. Nonetheless, the β2 mutant lines selected with WLW were sensitized by as much as 3- to 5-fold to EY 4–78, similar to WLL (Figures 2, S1A, and S3A; Tables S1 and S2).

Carmaphycin B, a naturally derived epoxyketone inhibitor, is known to exert potent and selective activity against the *Plasmodium* proteasome.^22^ We tested four carmaphycin B analogs, J-50, J-71, J-78, and J-80 (Figure 1), which share the same epoxyketone reactive group as the parent compound but show improved pharmacological properties.^44^ Like the vinyl sulfones, these compounds are also covalent, irreversible peptide inhibitors. All four epoxyketones are highly specific for β5. Our proteasome mutant lines showed nearly identical resistance profiles for all four epoxyketones (Figures 2, S1C, S1D, S3B, and S3C; Tables S1 and S2). Lines harboring β5 mutations exhibited the highest levels of resistance, exceeding increases observed with the vinyl sulfones, with all four epoxyketones showing 8- to 26-fold higher IC_50_ values against parasites expressing the β5 M45I and A20V mutations (selected with MPI-12 and MMV1579506, respectively). These data provide evidence of cross resistance between the epoxyketones and the boronate inhibitors MPI-12 and MMV1579506. The four epoxyketones showed 2- to 12-fold higher IC_50_ values against β6 mutant lines selected using the vinyl sulfone WLL or the previously published AsnEDA inhibitor PKS21004.^13^ In contrast, most epoxyketones showed lower IC_50_ values against WLW-selected β2 mutant lines.

We next tested two AsnEDA compounds, WHZ-04 and TDI-4528 (Figure 1), which, unlike the epoxyketones or vinyl sulfones, bind the proteasome in a non-covalent, reversible manner.^13,24,27^ Interestingly, the same two β5 mutations that conferred the highest degrees of resistance to the epoxyketones, namely A20V and M45I, also caused the most significant increases in WHZ-04 and TDI-4258 IC_50_ values. In fact, these boronate-selected A20V and M45I mutations conferred greater resistance to WHZ-04 and TDI-4258 than the β6 A117D mutation that was selected with a different AsnEDA, PKS21004. Parasites harboring a separate mutation at the same residue, β6 A117V (selected with WLL), became sensitized to TDI-4258. Hypersensitivity to WHZ-04 and TDI-4258 was also observed in all WLW-selected β2 mutant lines (Figures 2, S1E, and S1F; Tables S1 and S2).

We also tested two modified peptides, compounds 4 and 6 (Figure 1), which were previously identified as high-affinity, non-covalent inhibitors of the *Plasmodium* proteasome, targeting the β5 subunit.^20^ For these assays, we used three representative mutant lines harboring mutations in β2, β5, or β6, as restricted compound availability precluded additional testing. Both modified peptides exhibited identical resistance profiles, with the WLL-selected β5 A20S line displaying the highest levels of resistance, followed by the β6 A117V line (Figure 2). Similar to other compounds that primarily inhibit the β5 subunit (e.g., the epoxyketones and AsnEDA compounds), the WLW-selected β2 C31Y mutant line was the most susceptible to both peptide inhibitors (Figures S3D and S3E; Table S1).

Finally, we tested two commercially available agents designed to target the human proteasome, namely the boronate inhibitor bortezomib and the epoxyketone inhibitor epoxomicin (Figure 1). These inhibitors exhibited moderate or potent activity, respectively, against *P. falciparum* parasites, consistent with prior studies.^12,13,42^ The β5 M45I mutation, selected with MPI-12 (another boronate), conferred the highest level of resistance to bortezomib (Figures 2 and S3F; Tables S1 and S2). The WLL-selected β6 A117D mutation also resulted in increased bortezomib IC_50_ values. For epoxomicin, only the β6 S208L mutation resulted in a significant (3-fold) IC_50_ shift relative to the corresponding WT line (Figures 2 and S3G; Tables S1 and S2). These results suggest that epoxomicin is minimally affected by mutations that confer resistance to *Plasmodium*-selective proteasome inhibitors.

Although all of the compounds tested herein inhibit the catalytic β subunits of the 20S proteasome core particle, we have previously shown that mutations in the 19S regulatory particle of the 26S *P. falciparum* proteasome can also mediate resistance to the vinyl sulfone WLW.^29^ We tested whether three WLW-selected 19S mutants, namely RPT4 E380* (premature stop codon), RPT5 G319S, and RPN6 E266K, could mediate cross resistance to any of the other classes of *Plasmodium* proteasome inhibitors. Consistent with our previous study,^29^ none of the three 19S mutations resulted in significant increases in WLL IC_50_ values, whereas all three mutations yielded 2-fold lower IC_50_ values for the related vinyl sulfone EY 4–78 (Figures S4A–S4C; Table S3). Small (<2-fold) increases in IC_50_ values were also observed for the epoxyketone compounds J-50, J-71, J-78, and J-80 (Figures S4D–S4G). All three 19S mutations sensitized parasites to the AsnEDA compounds WHZ-04 and TDI-4258 (Figures S4H and S4I). Conversely, these mutants tended to be less sensitive to bortezomib and epoxomicin (Figures S4J and S4K). These results suggest that conformational changes imposed by mutations in the 19S regulatory particles may in some cases modulate parasite susceptibility to β subunit inhibitors.

### Genetic engineering of inhibitor-selected proteasome mutations reveals that mutations are sufficient to drive resistance

To validate the role of drug-selected proteasome β subunit mutations in conferring resistance irrespective of the parasite background, we developed a CRISPR/Cas9 system to edit select β5 mutations, namely A20S (selected for resistance to WLL), A20V (MMV1579506-selected), and M45I (MPI-12-selected), into Dd2 parasites (Figure S5A). Gene-edited parasites were then tested in a new set of 72 h dose-response assays, with the original drug-selected lines harboring the same mutations and their respective parental lines included as controls. Lines were tested against WLL (a covalent, irreversible vinyl sulfone), J-80 (a covalent, irreversible epoxyketone), and TDI-8304 (a non-covalent, reversible macrocyclic peptide). The latter was recently identified as a pharmacologically superior alternative to the AsnEDA TDI-4258.^27^ IC_50_ values based on dose-response assays showed that the gene-edited lines phenocopied the original drug-pressured lines across each class, confirming that the β5 mutations tested (A20S, A20V, and M45I) were causal for resistance on different genetic backgrounds. For WLL, the β5 A20S edited and selected lines both yielded an ~2.5-fold increase in the WLL IC_50_, whereas no increases were observed with the MPI-12-selected M45I mutation and the MMV1579506-selected A20V mutation in either the edited or the drug-pressured lines (Figures S5B and S2D; Table S4). For J-80 and TDI-8304, all three mutations afforded moderate to high-grade resistance in both the edited and the selected lines (Figures S5C, S5D, S2E, and S2F; Table S4).

### Conditional knockdowns of the β2 or β5 proteasome subunits sensitize parasites to *Plasmodium*-selective proteasome inhibitors

**Figure 3.**
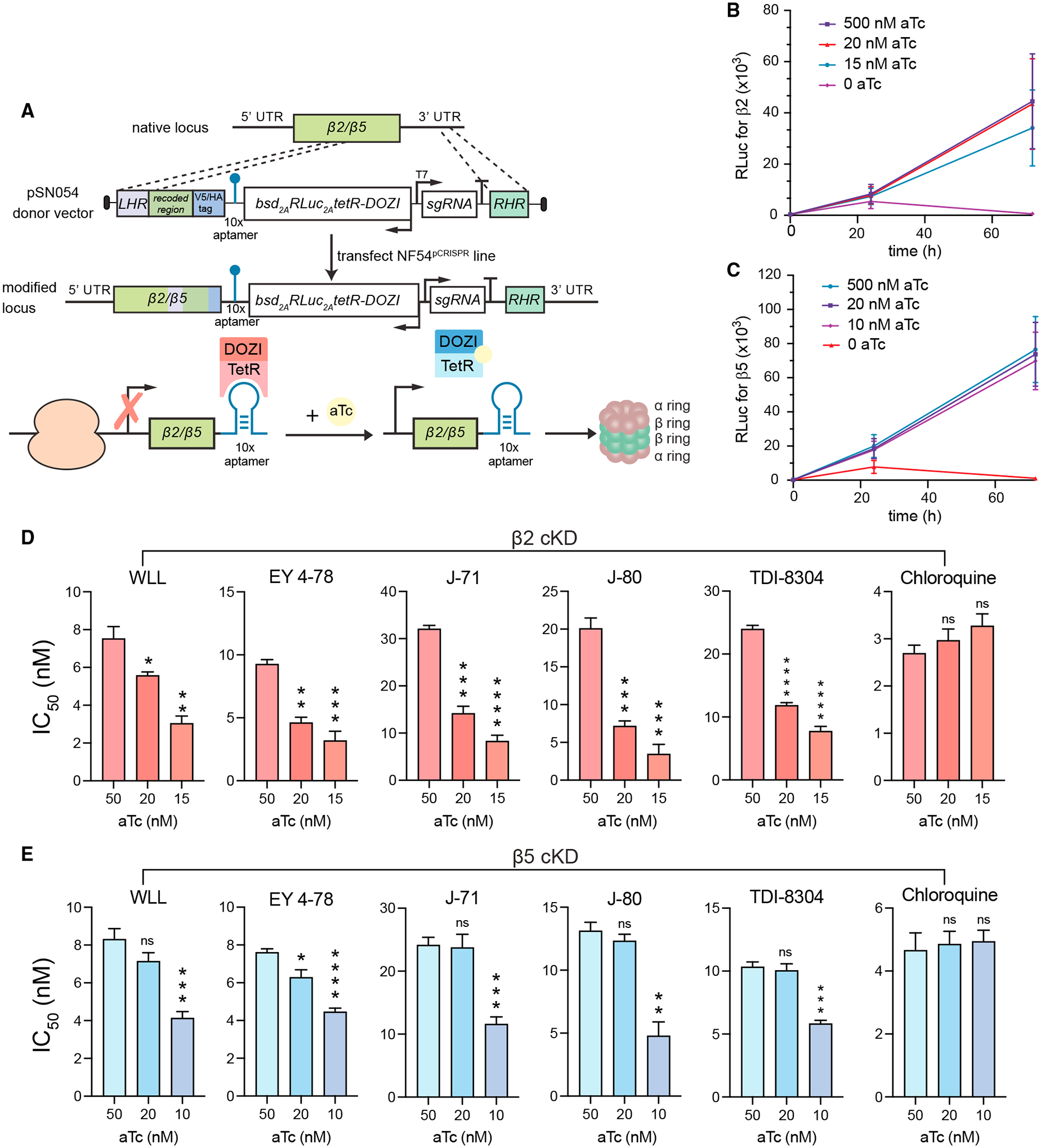
Regulation of Pf20S β2 and β5 expression by conditional knockdown sensitizes parasites to proteasome inhibition (A) Schematic of the pSN054 plasmid used to transfect NF54^pCRISPR^ parasites to generate conditional knockdowns (cKDs) of β2 and β5. The presence of 50 nM aTc allows for normal levels of protein translation. Removal of aTc blocks translation and reduces protein expression levels. The TetR-DOZI-T2A-RLuc-T2A-bsd cassette is expressed on the negative strand (see arrows indicating direction of transcription), with expression regulated by the *hsp86* 5′ untranslated region (UTR) sequence and the *hrp2* 3′ UTR sequence. For the proteasome β2 and β5 subunit genes, an *hsp86* 3′ UTR terminator sequence was inserted after the aptamer array. With this strategy, the 3′ end of the targets serve as the right homology arms for gene editing. (B and C) Growth rate data for (B) β2 and (C) β5 cKD lines cultured under a range of aTc concentrations. These cKD lines express an integrated Renilla luciferase cassette, enabling growth to be measured as a function of luciferase units (RLuc). These data show loss of parasite growth upon removal of aTc, consistent with these proteasome subunits being essential for parasite growth *in vitro*. Mean ± SD values were obtained from 3 independent experiments performed with technical duplicates. (D and E) IC_50_ values for (D) the transgenic β2 knockdown line and (E) the β5 knockdown line tested against representative proteasome inhibitors (WLL, EY 4–78, J-71, J-80, and TDI-8304) in decreasing concentrations of aTc. Chloroquine was used as a negative control. Results show mean IC_50_ ± SEM values from assays conducted on 4 to 5 independent occasions in duplicate (detailed in Table S5). Statistical significance was calculated using unpaired t tests with Welch’s correction, comparing parasites cultured at the permissive concentration of aTc (50 nM) with parasites cultured under knockdown conditions (10–20 nM aTc); *p < 0.05, **p < 0.01, ***p < 0.001, ****p < 0.0001; ns, not significant.

To further validate the *Plasmodium* proteasome as the target of our different classes of inhibitors, we used CRISPR/Cas9 to engineer conditional knockdown (cKD) parasites in an NF54 Cas9-expressing parasite line (denoted NF54^pCRISPR^). β5 or β2 expression levels were regulated via the TetR-DOZI system (Figure 3A).^50^ Basal expression levels were maintained by culturing parasites in the presence of 500 nM anhydrotetracycline (aTc). Medium or low expression levels were obtained for β5 cKD parasites by culturing in 20 or 10 nM aTc, respectively, and for β2 cKD parasites by culturing in 20 or 15 nM aTc, as these concentrations were found to reduce protein expression levels while retaining sufficient parasite growth. Western blot analysis of parasites harvested after 72 h validated protein-level knockdown of both subunits in the absence of aTc (Figures S6A and S6B). We also observed a significant growth defect in parasites cultured without aTc, consistent with the essentiality of these proteasome subunits (Figures 3B and 3C).

In the β2 cKD line, decreased levels of β2 were associated with decreased IC_50_ values for all proteasome inhibitors tested. At 15 nM aTc, we observed ~2- to 4-fold lower IC_50_ values for WLL (which targets β2 and β5), as well as for EY 4–78, J-71, J-80, and TDI-8304 (which primarily target β5). Lower IC_50_ values were also observed at 20 nM aTc (Figure 3D; Table S5). Control parasites cultured at 50 nM aTc, which allows for normal β5 expression, provided the reference values (we note that all of these compounds were more potent against NF54 parasites than the Cam3.II, Dd2, and V1/S lines tested above). Control assays with chloroquine showed no significant IC_50_ changes at the same aTc concentrations.

In the β5 cKD line, we also observed ~2-fold lower IC_50_ values for all inhibitors tested when cKD parasites were cultured at 10 nM aTc relative to parasites cultured at 50 nM (Figure 3E). No significant decreases were observed at 20 nM aTc. Chloroquine again showed no significant differences in IC_50_ values across aTc concentrations. Thus, although the compounds tested all selected for mutations in β5 and not β2, reduced expression levels of either β2 or β5 led to increased compound sensitivity, potentially because of a negative impact on proteasome complex assembly arising from lowered expression of either individual subunit.

### Irreversible *Plasmodium*-selective proteasome inhibitors display lower rates of *in vitro* resistance

**Figure 4.**
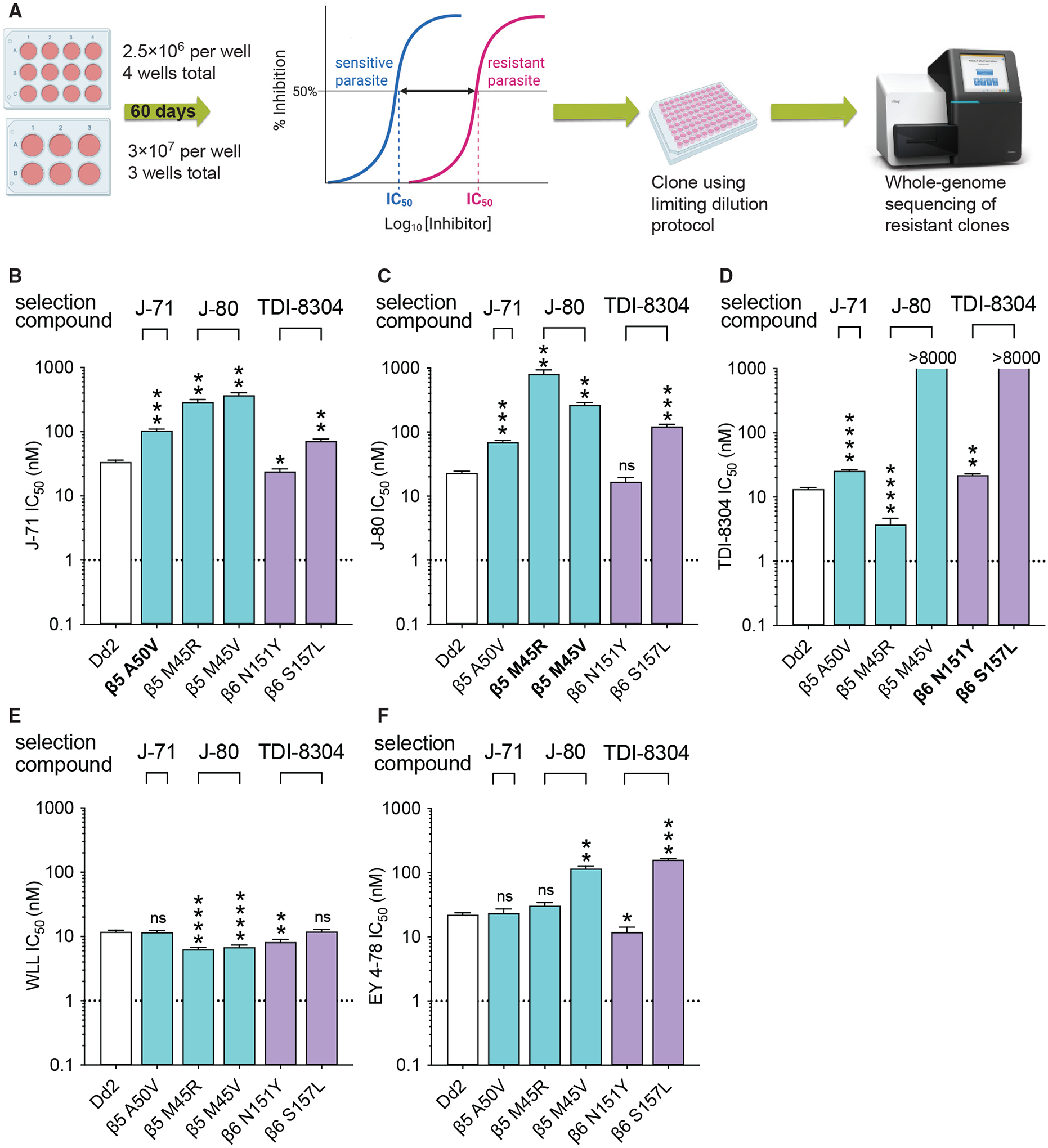
Minimum inoculum of resistance (MIR) selection experiments (A) Outline of MIR assay used to determine the lowest parasite starting inoculum required to select for resistance to a given compound. (B–F) IC_50_ values for selected mutants tested against J-80, J-71, TDI-8304, WLL, and EY 4–78. Lines shown in bold were selected for resistance to the test compound. Results show mean IC_50_ ± SEM values from assays conducted on 3 to 16 independent occasions in duplicate (detailed in Table S6). Fold changes are listed in Table S7. Statistical significance was calculated using unpaired t tests with Welch’s correction, comparing selected lines to the Dd2 parent; *p < 0.05, **p < 0.01, ***p < 0.001, ****p < 0.0001; ns, not significant.

Recent studies have identified resistance liabilities for several antimalarial compounds entering preclinical and human clinical trials, highlighting the need to identify compounds with low resistance risks early in the drug development process.^51^ Here, we used *in vitro* selection experiments with one or two representative compounds from the previously profiled classes of *Plasmodium*-selective inhibitors, including WLL, TDI-8304, J-71, and J-80, to directly compare resistance risks across chemotypes. To determine the minimum inoculum of resistance (MIR), we exposed WT Dd2 (clone B2) parasites to 3 × 3 IC_50_ concentrations of each compound throughout the selection process (single-step selection). Starting inocula were four wells at 2.5 × 10^6^ parasites and three wells at 3 × 10^7^ parasites (covering the range from 2.5 × 10^6^ to a total of 1 × 10^8^). Selections were maintained for 60 days or until recrudescence, and recrudescent parasites were cloned by limiting dilution (Figure 4A). For compounds that did not yield resistant parasites at these starting inocula, we performed an additional round of selections with three flasks, each with 3 × 10^8^ parasites (Table 2). Whole-genome sequencing of resistant clones revealed a series of novel β5 and β6 mutations (Table 2).

The MIR for the epoxyketones J-71 and J-80 were 1 × 10^8^ and 3 × 10^8^ parasites, respectively (Table 2). Resistance selections with J-71 yielded clones expressing an A50V mutation in β5, which resulted in low-level resistance (3-fold increase in IC_50_) to this compound as well as to J-80, and a 2-fold increase in the TDI-8304 IC_50_ (Figures 4B–4D; Table S6). For J-80, selections yielded clones with the β5 M45R or β5 M45V mutations, recalling the earlier M45I mutation observed in selections with the boronate MPI-12.^25^ Against J-71, β5 M45R and M45V lines exhibited 9- and 11-fold increases in IC_50_ levels, respectively (Figure 4B; Table S7). For J-80, we observed 35- and 12-fold increases in IC_50_ values for β5 M45R and M45V, respectively, compared with the 26-fold increase observed against the M45I line (Figures 2 and S1D; Table S7). These two selected mutants showed remarkably different profiles to the reversible inhibitor TDI-8304, which displayed a 4-fold lower IC_50_ against the β5 M45R line (indicating hypersentization), contrasting with an estimated 660-fold increase against the β5 M45V line (Table S7).

We next performed resistance selections with the non-covalent reversible inhibitor TDI-8304. This compound yielded resistant parasites with an MIR of 3 × 10^7^ (Table 2). Although this compound primarily inhibits β5, TDI-8304 selected for mutations in the β6 subunit, namely N151Y and S157L, which occur in residues that occupy the β5/β6 interface. Strikingly, the β6 S157L mutant line was 2,600-fold more resistant to TDI-8304 than its WT parent, while the β6 N151Y mutant yielded only a moderate (2-fold) increase in IC_50_ (Table S7). When tested against J-71, β6 S157L parasites were 2-fold more resistant, whereas β6 N151Y parasites were marginally more sensitive compared with the WT parental line. β6 S157L parasites were 5-fold more resistant to J-80, whereas the β6 N151Y mutation resulted in no change to the J-80 IC_50_ (Figures 4B and 4C; Table S7).

For WLL, we were unable to generate *in vitro* resistance with a total inoculum of up to 10^9^ parasites. This finding was consistent with our previously published results in which we obtained low-grade resistance to the vinyl sulfones WLL and WLW only with very large starting inocula of 2 × 10^9^ parasites.^29^

We then tested the J-71, J-80, and TDI-8304-selected lines against WLL and its optimized derivative EY 4–78. For WLL, all newly selected β5 or β6 mutations either resulted in no change in IC_50_ or yielded a 2-fold increase in susceptibility (Figure 4E; Table S7). Interestingly, when tested against EY 4–78, the β6 S157L and A117D mutations (selected with TDI-8304 and WLL, respectively) yielded modest (7- and 2-fold) increases in IC_50_ values, respectively (Figures 4F and S3). In contrast, the β6 N151Y line was 2-fold more sensitive to EY 4–78. The β5 M45V and M45I yielded ~5-fold higher EY 4–78 IC_50_ values, whereas no IC_50_ change was observed with the β5 M45R mutant.

### Molecular modeling of inhibitor-selected *Plasmodium* proteasome mutants

**Figure 5.**
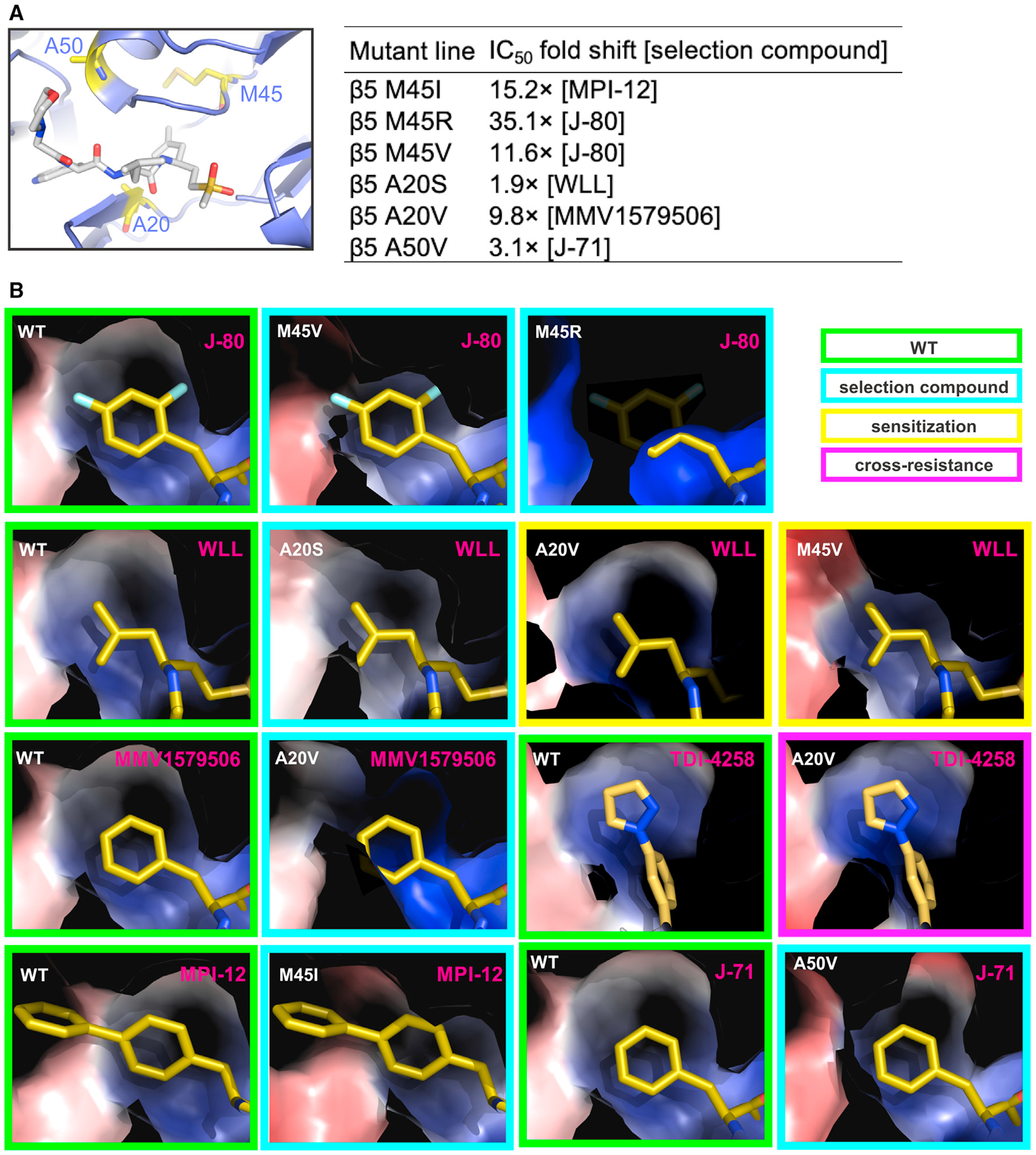
Molecular modeling of compound-selected *Plasmodium* proteasome mutations (A) Locations of the β5 A20, M45, and A50 residues (shown as sticks with yellow backbone) in the structure of the wild-type *Plasmodium* proteasome (shown as cartoon), with fitted WLL (cartoon with gray backbone).^21,29^ The table inset shows IC_50_ fold shifts for inhibitors tested against proteasome mutant lines compared with the wild-type parental line. Data for the β5 M45I line were taken from Xie et al.^25^ (B) Effects of the M45I, M45R, M45V, A20S, A20SV, and A50V mutations on the *Plasmodium* β5 P1 binding pocket. To facilitate comparison, the β5 P1 binding pocket of the wild-type proteasome (boxed in green) is shown in the same orientation as each of the selection mutants (cyan boxes). For all mutations, the proteasome models indicate that resistance to the selection compounds is primarily mediated by steric constraints that limit their access to the P1 binding site. Examples of sensitization (yellow boxes) and cross resistance (magenta boxes) are also shown. Protein models are represented as Van der Waals surfaces colored by electrostatic potential, overlaid with inhibitors.

We next used modeling to investigate the molecular basis for the resistance of *Plasmodium* proteasome mutants to their selection compounds. The modeling focused on mutations on the β5 M45, A20, and A50 residues, which were mapped onto the *Plasmodium* 20S proteasome structure (Figure 5A). The β5 M45 and A20 side chains are solvent exposed and directly face the β5 P1 binding pocket, suggesting that mutations on these residues could result in changes to compound binding properties without any significant protein conformational changes. However, the β5 A50 side chain is buried, and any mutations on this residue are more likely to lead to local conformational rearrangements associated with changes in intramolecular interactions, affecting the adjacent β5 P1 binding pocket.

Molecular dynamics algorithms are usually used for molecular modeling studies. However, such algorithms are computationally demanding and optimized for studies of small proteins, and thus are not suitable for modeling the full 20S proteasome complex. Previous modeling of *Plasmodium* proteasome β subunit mutations could be performed only by limiting the models to the two β subunits forming the ligand binding sites.^29^ Although informative, this required very careful supervision, as the lack of constraints imposed by the full protein-protein interactions that maintain the 20S proteasome assembly can easily lead to unrealistic model distortions. Here, we used the existing cryo-EM-derived structure of the WT *Plasmodium* 20S proteasome^21,29^ to create structural models of mutant complexes. These models (Figures 5A and 5B) clearly show that the inhibitor-selected β5 M45I, M45R, M45V, A20S, A20V, and A50V mutations all result in spatial contraction of the β5 P1 binding pocket that compromises the binding of each of the selection compounds. Resistance to these compounds can therefore be attributed primarily to steric constraints imposed by the proteasome mutations. Steric constraints were also associated with compounds for which cross resistance was observed, while sensitization was associated with changes in the electrostatic potential of protein surfaces lining the S1 binding pocket of the mutant proteasomes (Figure 5B).

## DISCUSSION

Combating ART-resistant *P. falciparum* is a key priority of malaria control and elimination efforts. Proteasome inhibitors have multistage antiplasmodial activity and display synergy with ART, making them attractive candidates for further drug development. Here we characterized a chemically diverse panel of *Plasmodium*-selective inhibitors, focusing on their resistance properties as a means to prioritize future lead optimization efforts. For these studies, we profiled a panel of covalent and non-covalent inhibitors, including vinyl sulfones, epoxyketones, and boronates in the former category, and AsnEDAs and reversible peptide inhibitors in the latter. By testing these inhibitors against mutant proteasome lines, we identified chemotype-specific mutations whose resistance profiles varied broadly between chemical classes, including instances of collateral sensitivity (Table 3).

Overall, the smallest IC_50_ shifts across our panel of mutant lines were observed with the vinyl sulfone inhibitors WLL, WLW, and EY 4–78 (up to 3-, 8-, and 2-fold, respectively). For these compounds, no mutations were found that caused IC_50_ increases as high as those that earlier proved problematic (30-fold or higher) in human clinical trials with inhibitors targeting dihydroorotate dehydrogenase (DHODH) or the sodium-dependent ATPase PfATP4.^30,32^ Unlike other classes of proteasome inhibitors, WLL and WLW exhibited compound- rather than class-specific patterns of resistance. This is most likely attributable to the fact that WLL simultaneously targets the β2 and β5 subunits of the Pf20S proteasome,^21^ while WLW primarily targets β2, and all other inhibitors tested herein primarily target β5. Consistent with prior reports, hypersensitization (or collateral sensitivity) to several compounds, including the dual-subunit targeting compound WLL, was mediated by WLW-selected mutations in the 20S β2 subunit. These data suggest that pairing inhibitors with specificity toward different proteasome subunits could serve as an effective tool to mitigate the potential emergence of resistance.

cKD of the β2 and β5 20S subunits sensitized parasites to representative compounds from all three classes of inhibitors examined, but not the control drug chloroquine. For β2, this result was likely the effect of a stoichiometric impact on proteasome assembly rather than direct targeting of this subunit by any of the compounds tested. Indeed, the effect of the β2 cKD was greater than that of the β5 knockdown, likely due to the increased sensitivity of parasites to reduced levels of β2 subunit expression.

MIR selections identified the vinyl sulfone WLL as a particularly refractory inhibitor, with no recrudescence observed at inocula up to 10^9^ asexual blood-stage parasites. These data substantiate our prior observation that resistance to vinyl sulfones is relatively difficult to achieve, requiring upwards of 2 × 10^9^ parasites.^29^ This compares very favorably with new antimalarials recently evaluated in patient exploratory trials, whose MIR values often range from 10^6^ to 10^9^ parasites, with the lower values associated with increased risk of treatment failure because of readily acquired resistance.^30,32,51^ We suspect that the low risk of resistance to WLL is associated with its binding to both the β2 and the β5 subunits.^21^

By comparison, selections with the epoxyketones J-71 and J-80 and the macrocyclic peptide TDI-8304 yielded MIR values ranging from 3 × 10^7^ (for TDI-8304) to 1 × 10^8^ and 3 × 10^8^ for J-71 and J-80, respectively. Resistance to these compounds was mediated by mutations in the β5 and β6 subunits. Interestingly, two of these mutations (M45V and M45R) occurred at a residue earlier found to mutate to M45I in response to selection with the potent boronate MPI-12.^25^ Our profiling of this M45I mutant line demonstrated that it mediates moderate resistance to several epoxyketone and AsnEDA inhibitors and lower-level cross resistance to the vinyl sulfone inhibitor EY 4–78. WLL and WLW did not lose potency against this mutant line. M45, located at the β5 S1 binding pocket, determines the interactions with the P1 residue of substrates and inhibitors. This residue’s methionine side chain is flexible and can project inward to accommodate the small, hydrophobic P1 moiety of some inhibitors, or outward for bulkier substrates. Mutation to an isoleucine or valine introduces larger, hydrophobic side chains that we predict would significantly interfere with binding of inhibitors with large P1 moieties. Modeling of compound-selected mutants of the *Plasmodium* 20S proteasome strongly supports the notion that resistance to the compounds tested results from mutation-imposed steric constraints at the β5 P1 binding pocket.

Our data illustrate that resistance profiles can differ even for compounds with similar binding modes, for example, vinyl sulfones and epoxyketones. Both are irreversible, covalent inhibitors, but the vinyl sulfones were markedly less prone to resistance compared to the epoxyketones. This may be due to the fact that some electrophiles are more sensitive to their positioning in the active site relative to the nucleophile, which in the case of the epoxyketones may make them more susceptible to point mutations that alter binding within the active site pockets and affect their ability to form covalent adducts. While our studies suggest that the vinyl sulfones have the most optimal properties for avoiding resistance, their resistance properties are highly compound-specific. Thus, it may be possible to tune the resistance properties of compounds in each of the different classes of inhibitors to minimize the liabilities for resistance generation.

Reversible binding inhibitors (i.e., AsnEDAs and TDI-8304) showed the highest IC_50_ shifts (up to 20- to 30-fold) when tested against proteasome mutant lines and were the most prone to acquiring resistance *in vitro*. This is likely due to the fact that reversible binding compounds reach an equilibrium of bound and unbound states that depends on the K_m_ values for binding. For these compounds, single point mutations can interrupt optimal binding. In the case of covalent compounds, when a similar drop in binding energy occurs, the compounds will bind less efficiently to the active site but will still eventually become covalently bound in place. Over time, covalent inhibitors can continue to block proteasome activity, making generation of resistance more difficult. Any highly significant changes to the proteasome that would effectively prevent inhibitor binding would also likely have an impact on the normal proteolytic function of the proteasome. Thus, our data provide additional support for the use of covalent inhibitors of the *Plasmodium* proteasome to suppress resistance mechanisms. We note that covalent irreversible inhibitors that are selective for parasite proteasomes have the caveat that any off-target binding to host proteasomes or other host proteases can carry an increased risk of toxicity compared with other types of inhibitors, requiring additional scrutiny during any further drug development efforts.^52,53^

For *P. falciparum*, we note that none of the proteasome mutations selected herein or previously obtained (Tables 2 and S2) were found recently in a sample of ~750 Ugandan isolates with sequenced β2 and β5 genes.^54^ That study identified a naturally occurring β2 S214F mutation that was associated with a 3- and 5-fold higher IC_50_ for the peptide boronates MMV1579506 and MPI-12 (also known as MMV1794229), respectively. The vinyl sulfones WLL and WLW as well as the AsnEDA TDI-4258 and the macrocyclic peptide TDI-8304 all retained full activity against the Ugandan isolates tested.

### Limitations of the study

One limitation of our study is that we profiled only four compounds in the MIR studies, because of the quantity of work required. Additional data would make for a more comprehensive assessment. Another limitation is that our structural modeling was restricted to a subset of β5 mutations and compounds. Additional modeling would provide more insight into the structural basis of resistance and collateral sensitivity. We also did not biochemically profile our compounds against enriched preparations of *P. falciparum* versus human proteasomes or use activity-based probe profiling to quantify the impact of 20S subunit mutations on inhibitor binding.^22,29^

Other crucial factors in drug development remain to be addressed before any *Plasmodium*-selective proteasome inhibitors can advance to human clinical trials. This includes generating orally bioavailable inhibitors, which in general is challenging for any peptide-based drug. Ultimately, the ideal candidate compound will likely combine features of several of the classes of inhibitors discussed herein while maintaining their activity across multiple parasite life-cycle stages and their unique and established property of synergizing with ART.

## STAR⋆METHODS

### RESOURCE AVAILABILITY

#### Lead contact

Further information and requests for resources and reagents should be directed to the lead contact, David Fidock (df2260@cumc.columbia.edu).

#### Materials availability

Please note that amounts of experimental compounds may be restricted and might require resynthesis. Chemical structures for the compounds used in these studies are shown in Figures 1 and S2A.

#### Data and code availability

- All datasets generated during this study are provided in separate spreadsheets as part of Tables S1–S7.
- No code was generated.
- Any additional information required to reanalyze the data reported in this paper is available from the lead contact upon request.

### EXPERIMENTAL MODEL AND SUBJECT DETAILS

Asexual blood-stage parasites were cultured at 3% hematocrit in RPMI-1640 medium supplemented with 50 μM hypoxanthine, 2.1 g/L NaHCO_3_, 2 mM L-glutamine, 25 mM HEPES, 0.5% (w/v) AlbuMAXII (Invitrogen) and 10 μg/mL gentamicin. Parasites were maintained at 37°C in modular incubator chambers supplied with a 5% CO_2_/5% O_2_/90% N_2_ gas mixture. Resistance selection and gene editing studies were performed using the Dd2-B2 clone,^56^ referred to herein as Dd2. De-identified human erythrocytes were sourced ethically from the Interstate Blood Bank (Memphis, TN) from anonymized blood donors, and their research use for cell culture was in accordance with terms of informed consent under a protocol approved by the Columbia University Medical Center Institutional Review Board, which designated this as not human subjects research.

### METHOD DETAILS

#### Compounds

Vinyl sulfone inhibitors (WLL (WLL-vs), WLW (WLW-vs) and EY 4–78) and N,C-capped peptides (Compounds 4 and 6) were synthesized by the Bogyo Lab.^20,21,23^ Asparagine ethylenediamine (AsnEDA) inhibitors (WHZ-04, TDI-4258) and the macrocyclic peptide TDI-8304 were synthesized by the Lin Lab.^13,24,27^ Epoxyketone inhibitors (J-50, J-71, J-78 and J-80) were synthesized by the Gerwick Lab.^44^ Synthetic methods and characterization data for all of these compounds are described in the articles cited above. Details for MMV1579506 are provided below. Bortezomib and epoxomicin were purchased from Millipore-Sigma.

#### Synthesis of *tert*-butyl N-[(5S)-5-[[(2R)-1-acetylpyrrolidine-2-carbonyl]amino]-6-oxo-6-[[(1R)-2-phenyl-1-[(1S,2S,6R,8S)-2,9,9-trimethyl-3,5-dioxa-4-boratricyclo[6.1.1.02,6]-decan-4-yl]-ethyl]amino]hexyl]carbamate (MMV1579506)


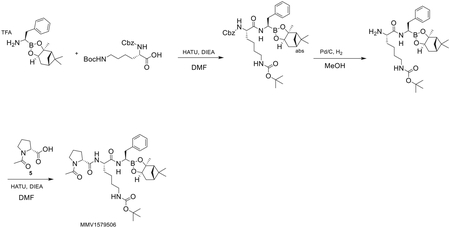


To a solution of (1R)-2-phenyl-1-[(1S,2S,6R,8S)-2,9,9-trimethyl-3,5-dioxa-4-boratricyclo-[6.1.1.02,6]decan-4-yl]ethanamine. trifluoroacetic acid salt (1.0 g, 2.41 mmol) in dimethyl formamide (15 mL) was added (2S)-2-{[(benzyloxy)carbonyl]amino}-6-{[(*tert*-butoxy)carbonyl]amino}hexanoic acid (1.09 g, 2.89 mmol) and hexafluorophosphate azabenzotriazole tetramethyl uronium (HATU, 1.09 g, 2.89 mmol). Di-isopropylethylamine (0.934 g, 7.23 mmol) was added at −50° C. The reaction mixture was stirred for 3 hr at 20° C. To the reaction mixture was added water (20 mL) at −50° C, after which a white solid was formed. The solid was collected by filtration, then the crude product was dissolved in EtOAc (30 mL) and the organic phase was washed with brine (20 mL × 2), dried over Na_2_SO_4_, filtered, and concentrated under vacuum to give *tert*-butyl N-[(5S)-5-(benzyloxycarbonylamino)-6-oxo-6-[[(1R)-2-phenyl-1-[(1S,2S,6R,8S)-2,9,9-trimethyl-3,5-dioxa-4-boratricyclo[6.1.1.02,6]decan-4-yl]ethyl]-amino]hexyl]carbamate (1.5 g, 89.3% yield). ^1^H NMR (400 MHz, CDCl_3_) δ 7.26 (m, 5 H), 7.17 (s, 2 H), 7.11 (br dd, *J*=12.1, 6.5 Hz, 3 H), 6.23 (br s, 1 H), 5.34 (br s, 1 H), 5.00 (s, 2 H), 4.58 (br s, 1 H), 4.23 (m, 1 H), 4.06 (m, 1 H), 3.12 (br d, *J*=4.5 Hz, 1 H), 2.99 (br s, 2 H), 2.90 (m, 1 H), 2.73 (m, 1 H), 2.25 (m, 1 H), 2.06 (m, 1 H), 1.93 (t, *J*=5.5 Hz, 1 H), 1.77 (m, 3H), 1.57 (br s, 1 H), 1.31 (br d, *J*=12.3 Hz, 16 H), 1.20 (m, 4 H), 0.77 (s, 3 H).

A mixture of *tert*-butyl N-[(5S)-5-(benzyloxycarbonylamino)-6-oxo-6-[[(1R)-2-phenyl-1-[(1S,2S,6R,8S)-2,9,9-trimethyl-3,5-dioxa-4-boratricyclo[6.1.1.02,6]decan-4-yl]ethyl]amino]-hexyl]carbamate (6.30 g, 9.52 mmol) and Pd/C (3.01 g) in methanol (80 mL) was placed under an atmosphere of H_2_ (created by vacuum evacuation and backfilling with H_2_ gas a total of three times). The mixture was stirred at 25° C under H_2_ (30 psi) for 2 hr then filtered over celite. The celite pad was rinsed well with methanol. The filtrate was then concentrated to dryness to give *tert*-butyl N-[(5S)-5-amino-6-oxo-6-[[(1R)-2-phenyl-1-[(1S,2S,6R,8S)-2,9,9-trimethyl-3,5-dioxa-4-boratricyclo-[6.1.1.02,6]decan-4-yl]ethyl]amino]hexyl]carbamate (4.70 g, 93.6% yield), ^1^H NMR (400 MHz, CDCl_3_) δ 7.33 (m, 6 H), 4.52 (m, 1 H), 4.24 (br d, *J*=8.1 Hz, 1 H), 3.47 (m, 1 H), 3.03 (m, 4 H), 2.70 (m, 1 H), 2.31 (m, 1 H), 2.06 (m, 2 H), 1.81 (m, 3 H), 1.38 (m, 23 H), 0.83 (m, 3 H).

To a solution of *tert*-butyl N-[(5S)-5-amino-6-oxo-6-[[(1R)-2-phenyl-1-[(1S,2S,6R,8S)-2,9,9-trimethyl-3,5-dioxa-4-boratricyclo [6.1.1.02,6]decan-4-yl]ethyl]amino]hexyl]carbamate (4.5 g, 8.53 mmol) and (2R)-1-acetylpyrrolidine-2-carboxylic acid (1.34 g, 8.53 mmol) in dicholormethane (40 mL) at 15° C was added propanephosphonic acid anhydride (T_3_P, 10.8 g, 17.0 mmol) and triethylamine (5.93 mL, 42.6 mmol). The solution was stirred for 3 hr at 15° C and then water (50 mL) and dicholormethane were added. The aqueous phase was extracted with dicholormethane (50 mL × 3), then the combined organic layers were washed with brine (30 mL), dried over Na_2_SO_4_, filtered and concentrated. The residue was purified by column chromatography (SiO_2_, elution with petroleum ether/EtOAc=3:1 to EtOH/EtOAc=10:1) to give *tert*-butyl N-[(5S)-5-[[(2R)-1-acetylpyrrolidine-2-carbonyl]amino]-6-oxo-6-[[(1R)-2-phenyl-1-[(1S,2S,6R,8S)-2,9,9-trimethyl-3,5-dioxa-4-boratri-cyclo[6.1.1.02,6]decan-4-yl]ethyl]amino]hexyl]carbamate (3.60 g, 63.3% yield). [α]_D_^22^ - 38 (*c* 0.1, CHCl_3_). ^1^H NMR (400 MHz, CDCl_3_) δ 7.27 (br d, J=7.6 Hz, 2 H), 7.18 (br t, J=7.3 Hz, 2 H), 7.10 (m, 1 H), 4.54 (td, J=8.8, 4.2 Hz, 1 H), 4.17 (br d, J=7.6 Hz, 2 H), 3.48 (m, 3H), 3.18 (br t, J=6.5 Hz, 1 H), 3.08 (br d, J=5.9 Hz, 2 H), 2.88 (qd, J=13.8, 7.6 Hz, 2 H), 2.15 (m, 4 H), 1.93 (m, 6 H), 1.82 (br s, 2 H), 1.73 (m, 2 H), 1.60 (s, 3 H), 1.42 (m, 13 H), 1.30 (s, 3 H), 1.20 (s, 3 H), 0.80 (s, 3 H). LC-MS (TFA): m/z = 667.5 (M+H).

#### ^1^H NMR for MMV1579506


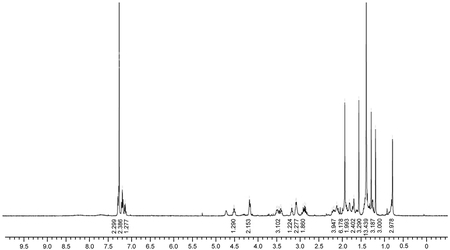


#### *In vitro* drug susceptibility assays

IC_50_ values for inhibitors against proteasome WT and mutant lines were determined by exposing parasites to serial dilutions of each compound in dose-response assays using asexual blood stage parasites. Compounds were tested in duplicate in 96-well plates, with the final volume per well equal to 200 μL. Parasites were seeded at 0.2% parasitemia and 1% hematocrit. After 72 h, parasites were stained with 1×SYBR Green and 100 nM MitoTracker Deep Red (ThermoFisher) and parasite viability was measured on an iQue Plus flow cytometer. IC_50_ values were derived by nonlinear regression (GraphPad Prism, version 9).

#### Genome editing

The A20S, A20V and M45I mutations in the 20S proteasome β5 (PF3D7_1011400) subunit were engineered into Dd2 parasites using a previously published “all-in-one” pDC2 CRISPR/Cas9 vector.^56^ This vector contains expression cassettes for *Cas9* (under control of the *calmodulin* promoter) and the selectable marker human dihydrofolate reductase (h*dhfr*) that confers resistance to WR99210 (under the *P. chabaudi dhfr-ts* (PcDT) promoter), as well as cloning sites for the insertion of a gene-specific guide RNA (gRNA) (under the *U6* promoter) and a gene-specific donor template for homology-directed repair (Table S7). β5-specific gRNAs (gRNA1 and gRNA2) were selected using the online tool ChopChop based on their proximity to the mutations of interest (A20S/V and M45I), guanine-cyto-sine (GC) content, and the absence of poly-adenine/thymine (A/T) tracts (http://chopchop.cbu.uib.no). gRNA primers were annealed and cloned into the pDC2 CRISPR/Cas9 vector using T4 ligase (NEB) at the BbsI restriction enzyme sites (Table S8). Donor fragments expressing mutations plus silent shield mutations at the gRNA1 and gRNA2 cut sites, or silent shield mutations alone (control), were synthesized by Genewiz, then cloned into the gRNA1 or gRNA2 pDC2 CRISPR/Cas9 vector by In-Fusion cloning (Takara) at the EcoRI/AatII sites. Final plasmids were confirmed by restriction digest and sequence verified. Plasmids were prepared for transfection using the NucleoBond^®^ Xtra midi prep kit (Macherey-Nagel).

Transfections were performed using a Bio-Rad electroporator as described previously.^57^ Each transfection employed 50 μg of plasmid DNA and 2.5 mL of predominantly ring-stage Dd2 parasites at ≥5% parasitemia and 3% hematocrit. Cultures were maintained in the presence of 2.5 nM WR99210 starting on day one post electroporation. Gene editing was assessed by Sanger sequencing of the 20S β5 locus, which was PCR-amplified from blood aliquots of bulk cultures.^58^ Edited parasite clones were obtained by limiting dilution cloning.

#### Generation of conditional knockdown parasite lines

Conditional knockdowns (cKDs) of the proteasome β2 (PF3D7_1328100) and β5 (PF3D7_1011400) subunits were generated by fusing the coding sequences of each gene with non-coding RNA aptamer sequences in the 3’ UTR, enabling translational regulation using the TetR/DOZI system.^50,55^ Gene editing was achieved by CRISPR/Cas9 using the linear pSN054 vector that contains cloning sites for the left homology region (LHR) and the right homology region (RHR), as well a gene-specific gRNA (under control of the *T7* promoter). RHRs for β2 and β5 were PCR-amplified from genomic DNA (gDNA). LHRs were synthesized using the BioXP^™^ 3200 System (SGI-DNA). For both β2 and β5, the region from the guide cut site to the 3’ end of the gene in the LHR was re-codonized to force integration of the RNA aptamer sequences. LHR and RHR fragments and gRNA sequences were cloned into the pSN054 linear vector by Gibson assembly (Table S7). LHRs were cloned upstream of the *blasticidin S-deaminase* selectable marker, the *Renilla luciferase* (*RLuc*) reporter, the 2A peptide-linked TetR-DOZI-RNA aptamer module, and C-terminal V5 and 2x-hemagglutinin (HA) tags. Final constructs were confirmed by restriction digest and sequence verified.

Transfections into Cas9- and T7 RNA polymerase-expressing NF54 parasites (NF54^pCRISPR^ line)^55^ were carried out by preloading erythrocytes with linearized vectors as previously described.^59^ Drug selection with 2.5 μg/mL of Blasticidin S (RPI Corp B12150-0.1) was initiated four days after transfection. Cell cultures were maintained in 500 nM anhydrotetracycline (aTc, Sigma-Aldrich). Parasite cell lines stably integrating the donor plasmids were monitored via Giemsa smears and RLuc measurements. Of note, 50 and 500 nM aTc were found to be equivalent in maintaining normal levels of protein expression.

#### Western blotting

To measure knockdown levels in the β2 and β5 cKD lines, parasites were cultured in 500 nM or 0 nM aTc to obtain normal or knocked-down expression levels, respectively. Parasite cultures were lysed after 72 h in 0.05% saponin and pellets were resuspended in lysis buffer consisting of 4% SDS and 0.5% Triton X-114 in 13PBS. Proteins were separated on a Mini-PROTEAN^®^ TGX^™^ Precast Gel (4–15% gradient) in Tris-Glycine buffer, transferred to a polyvinylidene fluoride (PVDF) membrane using the Mini Trans-Blot Electrophoretic Transfer Cell system according to the manufacturer’s instructions, and blocked with 100 mg/mL skim milk in TBS/Tween. Membrane-bound proteins were probed with mouse anti-HA (1:3,000; Sigma H3663) or rabbit anti-GAPDH (1:5,000; Abcam AB9485) primary antibodies, followed by anti-mouse (1:5,000; Thermo Fisher Scientific 62–6520) or anti-rabbit (1:5,000; Cell signaling 7074S) horseradish peroxidase (HRP)-conjugated secondary antibodies. Following incubation in SuperSignal^®^ West Pico Chemiluminescent substrate (Thermo Fisher Scientific PI34080), protein blots were imaged and analyzed using the ChemiDoc^™^ MP System and Image Lab 5.2.0 (Bio-Rad).

#### Growth assays

Assessment of parasite proliferation rates in β2 and β5 cKD lines upon knockdown of protein expression in varying aTc concentrations was carried out using luminescence as a readout for growth. Synchronous ring-stage parasites cultured in 0 nM aTc or increasing aTc concentrations (15, 20 or 500 nM for β2, and 10, 20 or 500 nM for β5) were set up in triplicate in 96-well plates and luminescence signals were taken at 0, 24, and 72 h post-invasion using the Renilla-Glo(R) Luciferase Assay System (Promega E2750) and the GloMax^®^ Discover Multimode Microplate Reader (Promega). Results were visualized using GraphPad Prism.

#### Minimum inoculum of resistance studies

Starting parasite inocula and MIR values for WLL, J-71, J-80 and TDI-8304 are listed in Table 2. For all compounds, resistance selections were performed by culturing Dd2 parasites continuously under 3×IC_50_ drug pressure. Drug-containing media was replaced every day for the first 6 days, then every 2 to 4 days. Red blood cells were replenished every week. Cultures were monitored by Giemsa staining and microscopy daily until parasites were cleared, then 2 to 3 times per week to detect recrudescence. Selections were maintained for 60 days or until recrudescent parasites were observed. Resistant clones were obtained from bulk cultures by limiting dilution cloning. The MIR value is defined as the minimum number of parasites used to obtain resistance and calculated as follows: total number of parasites inoculated ÷ total number of positive cultures. This formula includes lower inocula where there were no positive wells and excludes higher inocula in cases where lower inocula already yielded resistance. For WLL, no positive cultures were recovered at any inoculum and the MIR was > ((4×2.5×10^6^) + (3×3×10^7^) + (3×3×10^8^)), therefore >1×10^9^. The other MIR values were: for J-71 ((4×2.5×10^6^) + (3×3×10^7^))/1 = 1×10^8^; for J-80 ((4×2.5×10^6^) + (3×3×10^7^) + (3×3×10^8^))/3 = 3.3×10^8^; for TDI-8304 ((4×2.5×10^6^) + (3×3×10^7^))/3 = 3.3×10^7^.

#### Whole-genome sequencing

*P. falciparum* parasites were lysed in 0.05% saponin and washed with 1×PBS, and genomic DNA (gDNA) was purified using the QIAamp DNA Blood Midi Kit (Qiagen). gDNA concentrations were quantified by Qubit using the dsDNA HS Assay (Invitrogen). 200ng of gDNA was used to prepare sequencing libraries using the Illumina DNA Prep kit with Nextera^™^ DNA CD Indexes (Illumina). Samples were multiplexed and sequenced on an Illumina MiSeq using the MiSeq Reagent Kit V3 600 (Illumina) to obtain 300 base pair paired-end reads at an average of 30× depth of coverage. Sequence reads were aligned to the *P. falciparum* 3D7 reference genome (PlasmoDB version 48) using Burrow-Wheeler Alignment. PCR duplicates and unmapped reads were filtered out using Samtools and Picard. Reads were realigned around indels using GATK RealignerTargetCreator, and base quality scores were recalibrated using GATK BaseRecalibrator. GATK HaplotypeCaller (version 4.2.2) was used to identify all single nucleotide polymorphisms (SNPs). SNPs were filtered based on quality scores (variant quality as function of depth QD >1.5, mapping quality >40, min base quality score >18) and read depth (>5) to obtain high-quality SNPs, which were annotated using snpEFF. Integrated Genome Viewer was used to visually verify the presence of SNPs. BIC-Seq was used to check for copy number variations using the Bayesian statistical model.^60^ Copy number variations in highly polymorphic surface antigens and multi-gene families were removed as these are prone to stochastic changes during *in vitro* culture.

#### Molecular modeling of *Plasmodium* 20S proteasome mutants

The complete molecular models of the *Plasmodium* 20S proteasome mutations A20S/V, M45I/R/V, and A50V were built based on existing cryo-EM data. The mutated residues replaced the corresponding WT residues in the previously determined cryo-EM structure (PDB 5FMG) of the Pf20S proteasome. The resulting protein coordinates were optimized by real space refinement in Phenix^61^ using the *P. falciparum* proteasome cryo-EM map (EMD-3231^21^) as a template. The ligands were superimposed into the β5 P1 binding pockets based on existing structural data for the binding of related compounds to proteasome complexes (PDB 5FMG^21^, 4HRD^62^ and 7LXU^25^), without further fitting optimizations. For TDI-4258, the compound was fully built and docked into the proteasome models using AutoDockVina and UCSF Chimera.^63,64^ Graphic representations of all resulting models were prepared using the PyMOL Molecular Graphics System (Schrödinger).

### QUANTIFICATION AND STATISTICAL ANALYSIS

Details regarding statistical tests are reported in the legends to Figures 3, 4, and S1–S5, Tables S1 and S3–S6. Two-tailed unpaired Student *t* tests (with Welch’s correction) were employed throughout. Statistical analyses employed GraphPad Prism version 9.

## Supplementary Material

Supplementary Material

## Figures and Tables

**Table 1.** *Plasmodium falciparum* 26S proteasome wild-type and mutant lines assayed in this study

Line	Parental line	Proteasome complex	Proteasome subunit	Gene ID	Mutation preprocessing	Mutation postprocessing	Selection agent	Reference
β2 C31Y	Cam3.II K13^C580Y^	20S	β2	PF3D7_1328100	C72Y	C31Y	WLW	Stokes et al.^29^
β2 C31F	V1/S K13^wt^	20S	β2	PF3D7_1328100	C72F	C31F	WLW	Stokes et al.^29^
β2 A49E	V1/S K13^C580Y^	20S	β2	PF3D7_1328100	A90E	A49E	WLW	Stokes et al.^29^
β5 A20S	Cam3.II K13^C580Y^	20S	β5	PF3D7_1011400	A80S	A20S	WLL	Stokes et al.^29^
β5 A20V	Dd2 (clone B2)	20S	β5	PF3D7_1011400	A80V	A20V	MMV 1579506	This study
β5 M45I	Dd2 (clone B2)	20S	β5	PF3D7_1011400	M105I	M45I	MPI-12	Xie et al.^25^
β5 M45R	Dd2 (clone B2)	20S	β5	PF3D7_1011400	M105R	M45R	J-80	This study
β5 M45V	Dd2 (clone B2)	20S	β5	PF3D7_1011400	M105V	M45V	J-80	This study
β5 A49S	Dd2 (clone B2)	20S	β5	PF3D7_1011400	A109S	A49S	TDI-4258	Zhan et al.^24^
β5 A50V	Dd2 (clone B2)	20S	β5	PF3D7_1011400	A110V	A50V	J-71	This study
β6 A117V	V1/S K13^wt^	20S	β6	PF3D7_0518300	A117V	A117V	WLL	Stokes et al.^29^
β6 A117D	Dd2 (clone B2)	20S	β6	PF3D7_0518300	A117D	A117D	PKS21004	Kirkman et al.^13^
β6 N151Y	Dd2 (clone B2)	20S	β6	PF3D7_0518300	N151Y	N151Y	TDI-8304	This study
β6 S157L	Dd2 (clone B2)	20S	β6	PF3D7_0518300	S157L	S157L	TDI-8304	This study
β6 S208L	V1/S K13^C580Y^	20S	β6	PF3D7_0518300	S208L	S208L	WLL	Stokes et al.^29^
RPN6	Cam3.II K13^wt^	19S	–	PF3D7_1306400	E266K	E266K	WLW	Stokes et al.^29^
RPT5	Cam3.II K13^C580Y^	19S	–	PF3D7_1130400	G319S	G319S	WLW	Stokes et al.^29^
RPT4	Cam3.II K13^wt^	19S	–	PF3D7_1402300	E380*	E380*	WLW	Stokes et al.^29^

E380* refers to a premature stop-codon mutation.

**Table 2.** *P. falciparum* asexual blood-stage minimal inoculum for resistance (MIR) selection summary

Name	Chemical class	Starting selective pressure	Resistant parasites in 4 wells of 2.5 × 10^6^	Resistant parasites in 3 wells of 3 × 10^7^	Resistant parasites in 3 flasks of 3 × 10^8^	MIR	Selected proteasome mutation	IC_50_ fold shift against selection compound
WLL	vinyl sulfone	3 × IC_50_ (50.4 nM)	none	none	none	>1 × 10^9^	N/A	N/A
J-71	epoxyketone	3 × IC_50_ (152.4 nM)	none	1/3 wells	N/D	1 × 10^8^	β5 A50V	3.1×
J-80	epoxyketone	3 × IC_50_ (88.2 nM)	none	none	3/3 flasks	3 × 10^8^	β5 M45R, β5 M45V	35.1×, 11.6×
TDI-8304	macrocyclic peptide	3 × IC_50_ (33 nM)	none	3/3 wells	N/D	3 × 10^7^	β6 S157L, β6 N151Y	2,621×, 1.7×

IC_50_ fold shifts were calculated as the ratio of the IC_50_ of a compound against the selected mutant over its IC_50_ against the Dd2-B2 parental line. N, n = 3–20, 2. N/D, not done; N/A, not applicable. MIRs were calculated as the total number of parasites inoculated divided by the total number of positive cultures (when obtained). The formula extends up to the largest inoculum at which parasites were obtained (or tested in the case of negative cultures), and includes lower inocula (see STAR Methods).

**Table 3.** Summary of resistance properties of *P. falciparum* proteasome inhibitors

Name	Reference	Chemical class	Covalent/non-covalent	Reversible/non-reversible	Selectivity	Pf20S proteasome subunit target	MIR	Resistance-conferring mutations (reference)	Maximum resistance as fold change (mutation)	Maximum cross resistance as fold change (mutation and selection compound)	Collateral sensitivity observed
WLL	Li et al.^21^	vinyl sulfone	covalent	irreversible	*Plasmodium*	β5 and β2	>1× 10^9^	β5 A20S; β6 A117V, S208L (Stokes et al.^29^)	3.1× (β6 A117V)	1.1× (β6 A117D)	yes (β2 C31Y/F; β5 A20V, A49S)
WLW	Li et al.^21^	vinyl sulfone	covalent	irreversible	*Plasmodium*	β2	N/D	β2 C31F/Y, A49E; RPN6 E266K; RPT5 G319S; RPT4 E380* (Stokes et al.^29^)	8.3× (β2 C31F)	5.6× (β6 A117V, WLL)	yes (β5 A20V, M45I, A49S; β6 A117D)
EY 4–78	Yoo et al.^23^	vinyl sulfone	covalent	irreversible	*Plasmodium*	β5 and β2	N/D	–	–	5.2× (β5 M45I, MPI-12)	yes (β2 C31Y/F, A49E)
J-50	Almaliti et al.^44^	epoxyketone	covalent	irreversible	*Plasmodium*	β5	N/D	–	–	20× (β5 A20V, MMV1579506)	no
J-71	Almaliti et al.^44^	epoxyketone	covalent	irreversible	*Plasmodium*	β5	1 × 10^8^	β5 A50V (this study)	3.1× (β5 A50V)	14× (β5 A20V, MMV1579506)	yes (β2 C31Y)
J-78	Almaliti et al.^44^	epoxyketone	covalent	irreversible	*Plasmodium*	β5	N/D	–	–	21× (β5 A20V, MMV1579506)	yes (β2 C31Y)
J-80	Almaliti et al.^44^	epoxyketone	covalent	irreversible	*Plasmodium*	β5	3 × 10^8^	β5 M45 V/R (this study)	35× (β5 M45R)	26.4× (β5 M45I, MPI-12)	yes (β2 C31Y/F)
Epoxomicin	Czesny et al.^11^	epoxyketone	covalent	irreversible	host, *Plasmodium*	β5	N/D	–	–	4.1× (β6 A117V, WLL)	no
PK21004	Kirkman et al.^13^	AsnEDA	non-covalent	reversible	*Plasmodium*	β5	N/D	β6 A117D (Kirkman etal.^13^)	130× (β6 A117D)	N/D	N/D
WHZ-04	Zhan et al.^24^	AsnEDA	non-covalent	reversible	*Plasmodium*	β5	N/D			418× (β5 A20V, MMV1579506)	yes (β2 C31Y/F, A49E; β6 S208L)
TDI-4258	Zhan et al.^24^	AsnEDA	non-covalent	reversible	*Plasmodium*	β5	N/D	A49S (Zhan et al.^24^)	N/D	>300× (β5 A20V, MMV1579506)	yes (β2 C31Y/F, A49E; β6 A117V, S208L)
TDI-8304	Zhan et al.^27^	macrocyclic peptide	non-covalent	reversible	*Plasmodium*	β5	3 × 10^7^	β6 S157L, N151Y	2,621× (β6 S157L)	662× (β5 M45V, J-80)	yes (β5 M45R)
Compound 4	Li et al.^20^	N,C-capped peptide	non-covalent	reversible	*Plasmodium*	β5	N/D	–	–	5.2× (β5 A20S, WLL)	yes (β2 C31Y)
Compound 6	Li et al.^20^	N,C-capped peptide	non-covalent	reversible	*Plasmodium*	β5	N/D	–	–	4.5× (β5 A20S, WLL)	yes (β2 C31Y)
MMV1579506	Takeda	boronate	covalent	reversible	*Plasmodium*	β5	N/D	β5 A20V (this study)	9.8× (β5 A20V)	N/D	N/D
MPI-12	Xie et al.^25^	boronate	covalent	reversible	*Plasmodium*	β5	N/D	β5 M45I (Xie et al.^25^)	15× (β5 M45I)	N/D	N/D
Bortezomib	Xie et al.^42^	boronate	covalent	reversible	host, *Plasmodium*	β5	N/D	β5 M45I, L53F (Xie et al.^42^)	18× (β5 M45I)	38× (β6 A117V, WLL)	no

N/D, not done.

**Table:** KEY RESOURCES TABLE

REAGENT or RESOURCE	SOURCE	IDENTIFIER
Antibodies		
Mouse monoclonal anti-HA	Sigma-Aldrich	Cat. #H9658; RRID: AB_260092
Bacterial strains		
*E. coli* HST08 (Stellar Competent Cells)	Takara	Cat. #636766
Biological samples		
See below (cell lines)	N/A	N/A
Chemicals, peptides, and recombinant proteins		
All tested antimalarials and their structures are available in Figures 1 and S2.		
WLL	Bogyo Lab, Stanford University	N/A
WLW	Bogyo Lab, Stanford University	N/A
EY 4–78	Bogyo Lab, Stanford University	N/A
WHZ-04	Lin Lab, Weill Cornell Medicine	N/A
TDI-4258	Lin Lab, Weill Cornell Medicine	N/A
TDI-8304	Lin Lab, Weill Cornell Medicine	N/A
J-50	Gerwick Lab, University of California San Diego	N/A
J-71	Gerwick Lab, University of California San Diego	N/A
J-78	Gerwick Lab, University of California San Diego	N/A
J-80	Gerwick Lab, University of California San Diego	N/A
Compound 4	Bogyo Lab, Stanford University	N/A
Compound 6	Bogyo Lab, Stanford University	N/A
Bortezomib	Millipore Sigma	Cat. #5043140001
Epoxomicin	Millipore Sigma	Cat. #E3652-50UG
Chloroquine	Sigma-Aldrich	Cat. #C6628-25G
SYBR Green	Thermo Scientific	Cat. #S7563
MitoTracker Deep Red	Thermo Scientific	Cat. #M22426
Anhydrotetracycline	Sigma-Aldrich	Cat. #37919
WR99210	Jacobus Pharmaceuticals	N/A
Critical commercial assays		
In-Fusion HD Cloning Plus kit	Takara	Cat. #638909
Renilla-Glo(R) Luciferase Assay System	Promega	Cat. #E2750
Experimental models: Cell lines		
*P. falciparum* line Dd2	The Malaria Research and Reference Reagent Resource Center (MR4), BEI Resources	Dd2 (clone B2)
*P. falciparum* line Cam 3.II K13^WT^	Fidock Lab (Straimer et al.)^65^	Cam 3.II K13^wt^
*P. falciparum* line Cam 3.II K13^C580Y^	Fidock Lab (Straimer et al.)^65^	Cam 3.II K13^C580Y^
*P. falciparum* line V1/S K13^WT^	Fidock Lab (Straimer et al.)^65^	V1/S K13^wt^
*P. falciparum* line V1/S K13^C580Y^	Fidock Lab (Straimer et al.)^65^	V1/S K13^C580Y^
*P. falciparum* line NF54^pCRISPR^	Niles Lab (Nasamu et al.)^55^	NF54p^CRISPR^
Oligonucleotides		
See Table S7		
Recombinant DNA		
pDC2-cam-coSpCas9-U6-gRNA-hdhfr	Fidock Lab	pDC2-cam-coSpCas9-U6-gRNA-hdhfr
pSN054	Niles Lab (Nasamu et al.)^55^	pSN054
Software and algorithms		
GraphPad Prism Version 8	GraphPad Software, San Diego, CA, USA	www.graphpad.com
PyMOL Molecular Graphics System Version 2.5	Schrödinger	https://pymol.org/2

## Highlights

- Multiple proteasome-specific chemotypes can potently inhibit *P. falciparum* growth
- Resistance can arise via mutations in the β2, β5 or β6 subunits of the 20S proteasome core particle, or in the 19S regulatory particle
- WLL has a minimal resistance risk, attributable to its covalent binding to both β2 and β5
- Resistance to some chemotypes can produce collateral sensitivity to other compounds

## SIGNIFICANCE

Malaria’s impact on intertropical regions is unrelenting, with an estimated 619,000 deaths in African children below 5 years of age in 2021. Effective treatment with first-line artemisinin-based combination therapies is threatened by artemisinin-resistant parasites that are prevalent in Southeast Asia and are spreading rapidly across eastern Africa. *P. falciparum*-specific proteasome inhibitors are important assets in the pipeline for new antimalarial drugs as they display the ability to synergize with artemisinin derivatives, including against artemisinin-resistant parasites. These inhibitors bind the catalytic subunits of the proteasome, thereby preventing this multi-subunit complex from reducing artemisinin-induced proteotoxic stress by degrading damaged proteins. Here, we tested whether representatives of the leading chemical classes of *Plasmodium*-selective proteasome inhibitors differed in their propensity to select for drug-resistant parasites. These compounds differ in their chemical structures and modes of binding. We also assessed the degree to which mutations in the β2 or β5 subunit or proteasome accessory proteins mediated resistance and examined cross-resistance patterns. Our results identify the tripeptide vinyl sulfone WLL as having the most favorable profile, exhibiting a low risk of selecting for resistance and sustained potency against a panel of proteasome mutants. We attribute this feature to the covalent nature of this inhibitor and its irreversible dual binding of the β2 and β5 subunits. Conditional knockdown parasites confirmed compound selectivity for the 20S proteasome. We also identified proteasome mutations that resulted in collateral hypersensitivity, meaning that resistance to one inhibitor caused increased parasite susceptibility to another, creating a potential for resistance-refractory inhibitor combinations. Molecular modeling identified steric constraints in the mutated β5 P1 binding pockets that could reduce drug binding and account for parasite resistance. Our data provide compelling justification for further advancement of proteasome inhibitors with the goal of developing synergistic drug partners to treat artemisinin-resistant malaria.

## INCLUSION AND DIVERSITY

We support inclusive, diverse, and equitable conduct of research.
