# Supplementary material for: Mitigating the risk of antimalarial resistance via covalent dual-subunit inhibition of the Plasmodium proteasome
Source: Cell Chem Biol. Author manuscript; Available in PMC 2023 May 20. (PMC10198959; doi:10.1016/j.chembiol.2023.03.002)
Supplement: Supplementary file 1 [file NIHMS1895071-supplement-Supplementary_Material.pdf]

Cell Chemical Biology, Volume *30*

Supplemental information

Mitigating the risk of antimalarial resistance via covalent dual-subunit inhibition of the *Plasmodium* proteasome

Ioanna Deni, Barbara H. Stokes, Kurt E. Ward, Kate J. Fairhurst, Charisse Flerida A. Pasaje, Tomas Yeo, Shirin Akbar, Heekuk Park, Ryan Muir, Daniella S. Bick, Wenhu Zhan, Hao Zhang, Yi Jing Liu, Caroline L. Ng, Laura A. Kirkman, Jehad Almaliti, Alexandra E. Gould, Maëlle Duffey, Anthony J. O'Donoghue, Anne-Catrin Uhlemann, Jacquin C. Niles, Paula C.A. da Fonseca, William H. Gerwick, Gang Lin, Matthew Bogyo, and David A. Fidock

Figure S1 (related to Figures 1 and 2; Table 1; Tables S1 and S2). **Profiling of Pf20S β2, β5 and β6 subunit mutants against *Plasmodium*-selective proteasome inhibitors** page 2

Figure S2 (related to Table 1)**. Resistance to the boronate inhibitor MMV1579506 is mediated by the 20S β5 A20V mutation** page 4

Figure S3 (related to Figures 1 and 2; Table 1; and Tables S1 and S2). **Profiling of 20S beta subunit mutants against selective and non-selective *Plasmodium* proteasome inhibitors** page 6

Figure S4 (related to Table 1 and Table S3). **Profiling of 19S regulatory particle mutants against *Plasmodium*-selective and non-selective proteasome inhibitors** page 8

Figure S5 (related to Table 3). **CRISPR/Cas9-mediated editing of *P. falciparum* 20S β5 proteasome mutations** page 10

Figure S6 (related to Figure 3 and Table S5). **Conditional knock-down parasites demonstrate essentiality for proteasome β2 and β5 subunits** page 12

Table S1 (related to Figure 1; Figures S1 and S3). **EC_{50} values (nM) for inhibitor-selected proteasome mutant lines and proteasome wild-type controls** page 13

Table S2 (related to Figure 2; Table S1; Figure S1 and S3)**. EC_{50} fold shifts (nM) for inhibitor-selected proteasome mutant lines relative to their respective parental lines** page 15

Table S3 (related to Table 1 and Figure S4). **EC_{50} values (nM) for inhibitor-selected proteasome mutant lines and proteasome wild-type controls** page 16

Table S4 (related to Table 3 and Figure S5)**. EC_{50} values (nM) for CRISPR/Cas9-edited proteasome mutant lines compared with selected lines and proteasome wild-type controls** page 17

Table S5 (related to Figure 3). **EC_{50} values (nM) for 20S proteasome β2 and β5 conditional knockdown lines** page 18

Table S6 (related to Figure 4). **EC_{50} values (nM) for inhibitor-selected proteasome mutant lines and proteasome wild-type control** page 18

Table S7 (related to Figure 4 and Table S6). **IC_{50} fold shifts (nM) for selected proteasome mutant lines relative to their parental line Dd2-B2** page 19

Table S8 (related to Figure 3 and Figure S5)**. Oligonucleotides employed in this study** page 20

Supplementary References page 20

Figure S1

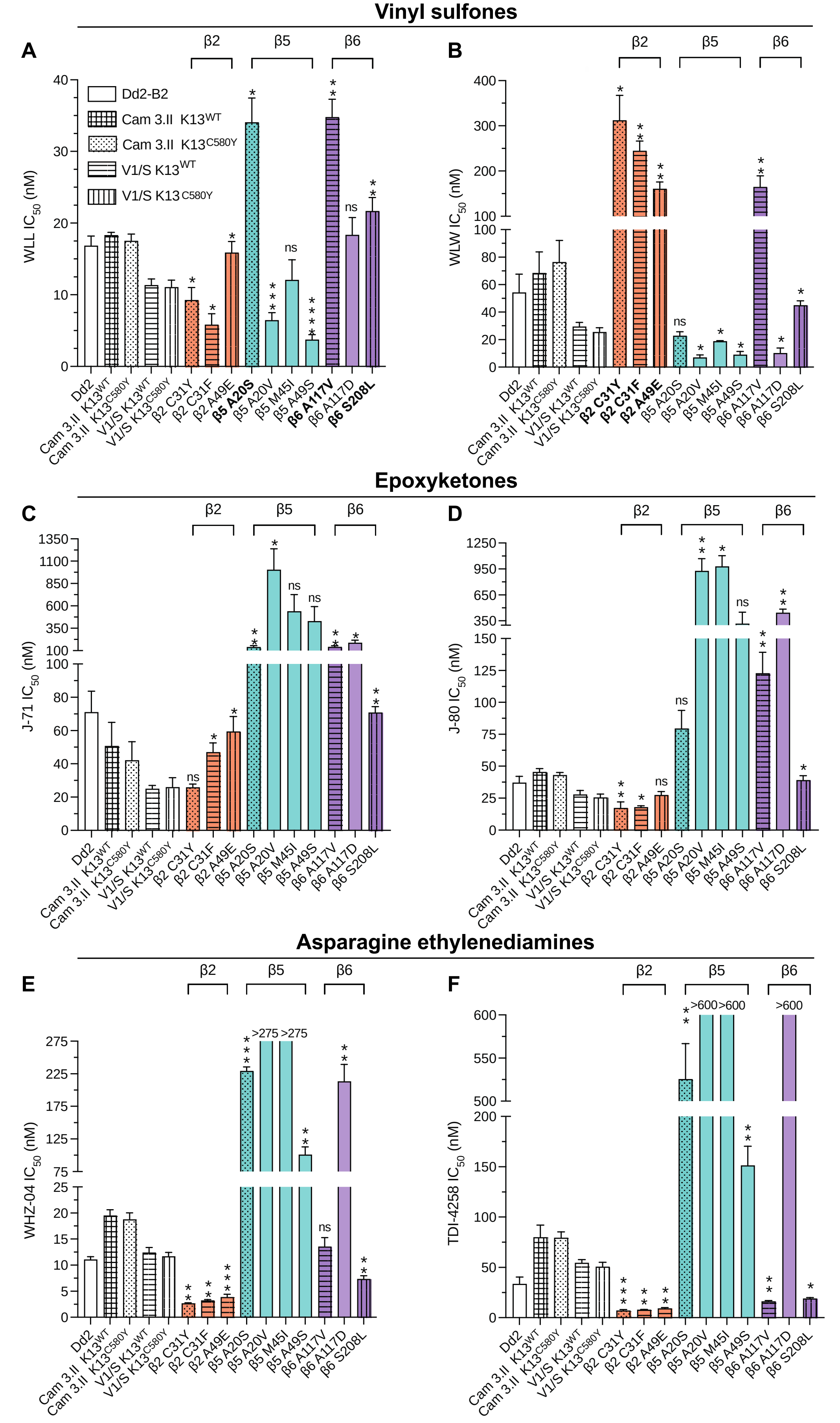

Vinyl sulfones
A
B
WLL IC50 (nM)
WLW IC50 (nM)
Dd2-B2
Cam 3.II K13WT
Cam 3.II K13C580Y
V1/S K13WT
V1/S K13C580Y
β2
β5
β6
Epoxyketones
C
D
J-71 IC50 (nM)
J-80 IC50 (nM)
Asparagine ethylenediamines
E
F
WHZ-04 IC50 (nM)
TDI-4258 IC50 (nM)
>275 >275
>600 >600
>600
Dd2
Cam 3.II K13WT
Cam 3.II K13C580Y
V1/S K13WT
V1/S K13C580Y
β2 C31Y
β2 C31F
β2 A49E
β5 A20S
β5 A20V
β5 M45I
β5 A49S
β6 A117V
β6 A117D
β6 S208L
ns

Figure S1. Profiling of Pf20S β2, β5 and β6 subunit mutants against *Plasmodium*-selective proteasome inhibitors. (A-F) IC_{50} values for *P. falciparum* 20S proteasome wild-type lines (Dd2, Cam3.II $K13^{WT}$, Cam3.II $K13^{C580Y}$, V1/S $K13^{WT}$ and V1/S $K13^{C580Y}$) and β subunit mutant lines tested against a panel of *Plasmodium*-selective inhibitors in 72 h dose-response assays. Cam3.II $K13^{WT}$ and Cam3.II $K13^{C580Y}$ are isogenic and differ solely at the artemisinin resistance locus *K13*, as are V1/S $K13^{WT}$ and V1/S $K13^{C580Y}$. For all compounds tested, the K13 C580Y mutation did not affect parasite susceptibility to proteasome inhibition. Compounds tested included: **(A, B)** vinyl sulfones (WLL and WLW), **(C, D)** epoxyketones (J-71 and J-80), and **(E, F)** asparagine ethylenediamines (WHZ-04 and TDI-4258). Lines indicated in bold were obtained from resistance selection studies with the compound being tested. Results show means ± SEM from 3 to 4 independent assays conducted in duplicate (see **Table S1**). Statistical significance was calculated using unpaired *t* tests with Welch's correction, comparing selected lines to their respective parental lines (detailed in **Table 1** and indicated here using hatching patterns specific to each background strain). *p<0.05; **p<0.01; ***p<0.001; ****p<0.0001; ns, not significant.

Figure S2

A **B**

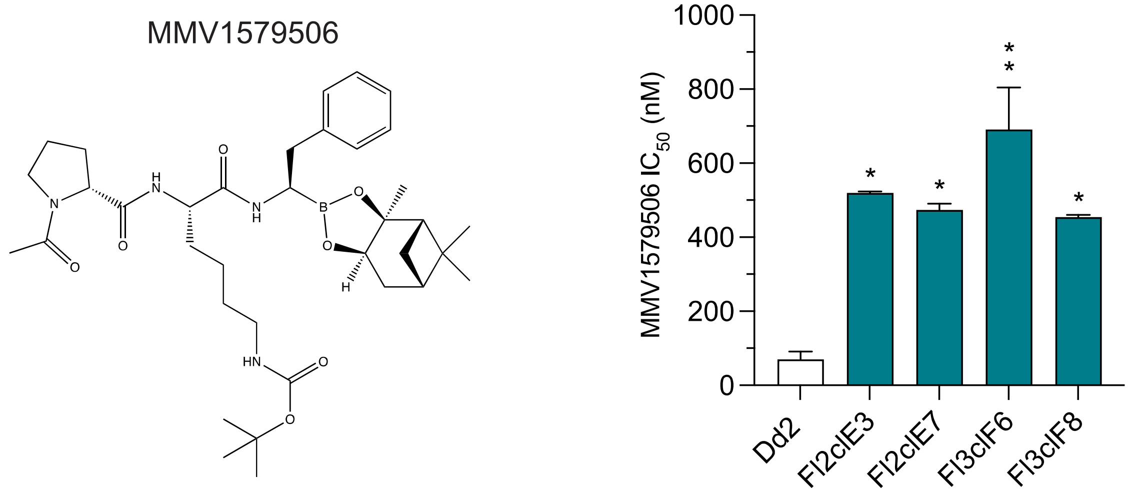

C

Name	Chemical Class	Starting Selective Pressure	Recrudescence	Selected Proteasome Mutation	IC_{50} fold shift against selection compound
MMV1579506	boronate	3×IC_{50} (70 nM)	3/3 flasks at 2×10^9/flask	A20V	9.8×

D **E** **F**

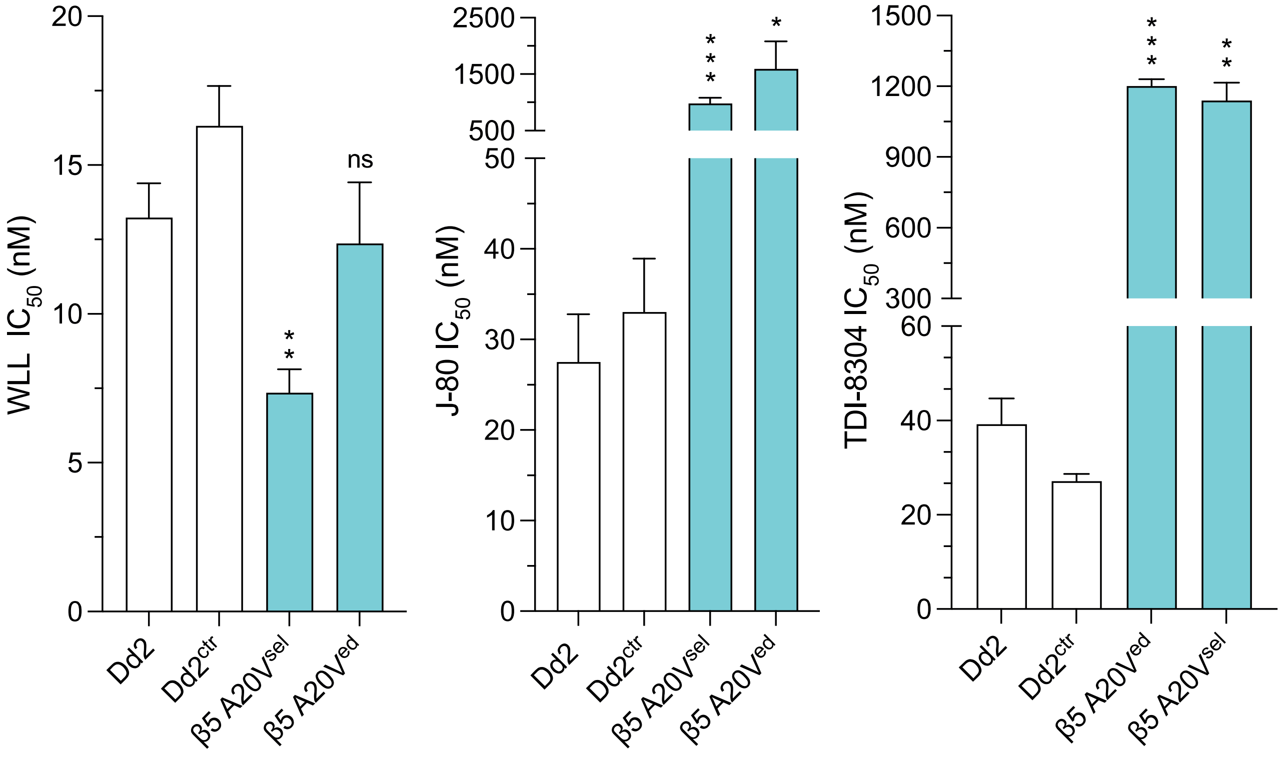

Figure S2. Resistance to the boronate inhibitor MMV1579506 is mediated by the 20S β5 A20V mutation. (A) Chemical structure of MMV1579506, a reversible, covalent boronate inhibitor. **(B)** IC_{50} values for *P. falciparum* Dd2 clones generated from MMV1579506 *in vitro* resistance selections tested against the selection compound. Whole-genome sequencing of MMV1579506-selected clones (from two separate flasks, Fl2 and Fl3, each inoculated with 2×10^9 parasites) revealed a β5 A20V mutation in all four clones. This mutation was also present in all three bulk cultures from the recrudescent flasks. Selected lines harboring the A20V mutation exhibited up to 9.8-fold increases in their IC_{50} values relative to the wild-type Dd2 parental line. **(C)** Summary of MMV1579506 selections that yielded resistant parasites. All three flasks inoculated at 2×10^9 parasites were positive. Separately, all 4 wells inoculated with 2.5×10^6 parasites per well and all 3 wells inoculated at 2×10^7 parasites/well remained negative (not shown). **(D-F)** IC_{50} values for Dd2 and drug-selected or CRISPR/Cas9 gene-edited β5 A20V lines (β5 $A20V^{sel}$ and β5 $A20V^{ed}$, respectively) tested against **(D)** WLL, **(E)** J-80, or **(F)** TDI-8304 in 72 h dose-response assays. The Dd2 control (ctr) line expresses silent binding-site mutations at the CRISPR/Cas9 cut site. Results show means ± SEM from assays conducted on 3 to 5 independent occasions in duplicate (see **Table S2**). Statistical significance was calculated using unpaired *t* tests with Welch's correction, comparing selected or edited lines to the Dd2 parent. *$p<0.05$; **$p<0.01$; ***$p<0.001$; ****$p<0.0001$; ns, not significant.

Figure S3

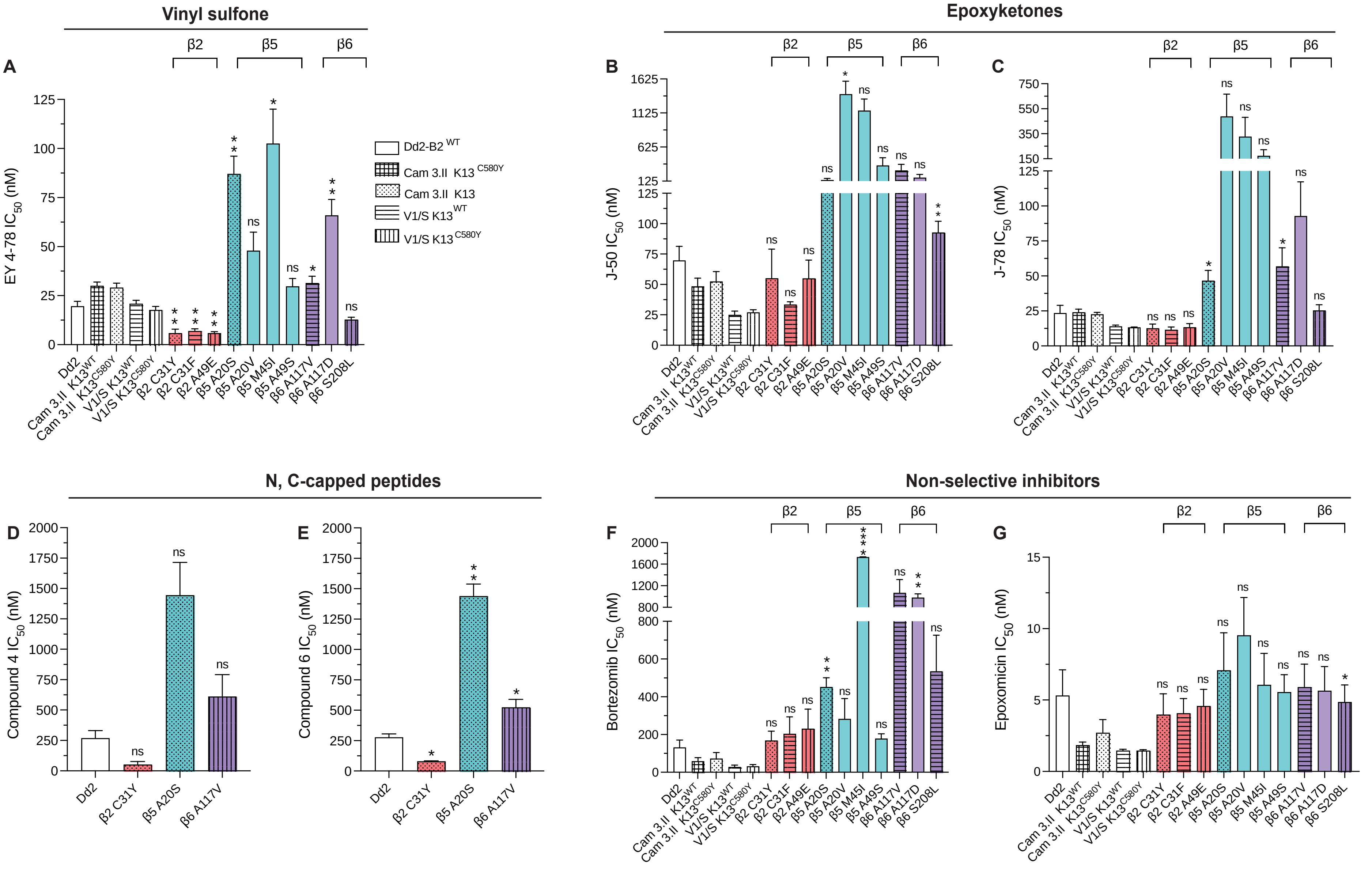

Vinyl sulfone
Epoxyketones
β2
β5
β6
A
B
C
EY 4-78 IC50 (nM)
J-50 IC50 (nM)
J-78 IC50 (nM)
Dd2-B2 WT
Cam 3.II K13 C580Y
Cam 3.II K13
V1/S K13 WT
V1/S K13 C580Y
Dd2
Cam 3.II K13WT
Cam 3.II K13C580Y
V1/S K13WT
V1/S K13C580Y
β2 C31Y
β2 C31F
β2 A49E
β5 A20S
β5 A20V
β5 M45I
β5 A49S
β6 A117V
β6 A117D
β6 S208L
ns
N, C-capped peptides
Non-selective inhibitors
D
E
F
G
Compound 4 IC50 (nM)
Compound 6 IC50 (nM)
Bortezomib IC50 (nM)
Epoxomicin IC50 (nM)

Figure S3. Profiling of 20S beta subunit mutants against selective and non-selective *Plasmodium* proteasome inhibitors. IC_{50} values for *P. falciparum* 20S proteasome wild-type lines (Dd2, Cam3.II $K13^{WT}$, Cam3.II $K13^{C580Y}$, V1/S $K13^{WT}$ and V1/S $K13^{C580Y}$) and mutant lines tested against additional *Plasmodium*-selective and non-selective inhibitors in 72 h dose-response assays, as described in **Figure 1**. Selective compounds tested included **(A)** EY 4-78 (vinyl sulfone), **(B, C)** J-50 and J-78 (epoxyketones), and **(D, E)** Compounds 4 and 6 (N,C-capped peptides). Non-selective inhibitors included **(F)** bortezomib and **(G)** epoxomicin. Results show means ± SEM from 3 to 4 independent assays conducted in duplicate (see **Table S1**). Statistical significance was calculated using unpaired *t* tests with Welch's correction, comparing selected lines to their respective parental lines (detailed in **Table 1** and indicated here using hatching patterns specific to each background strain). *p<0.05; **p<0.01; ***p<0.001; ****p<0.0001; ns, not significant.

Figure S4

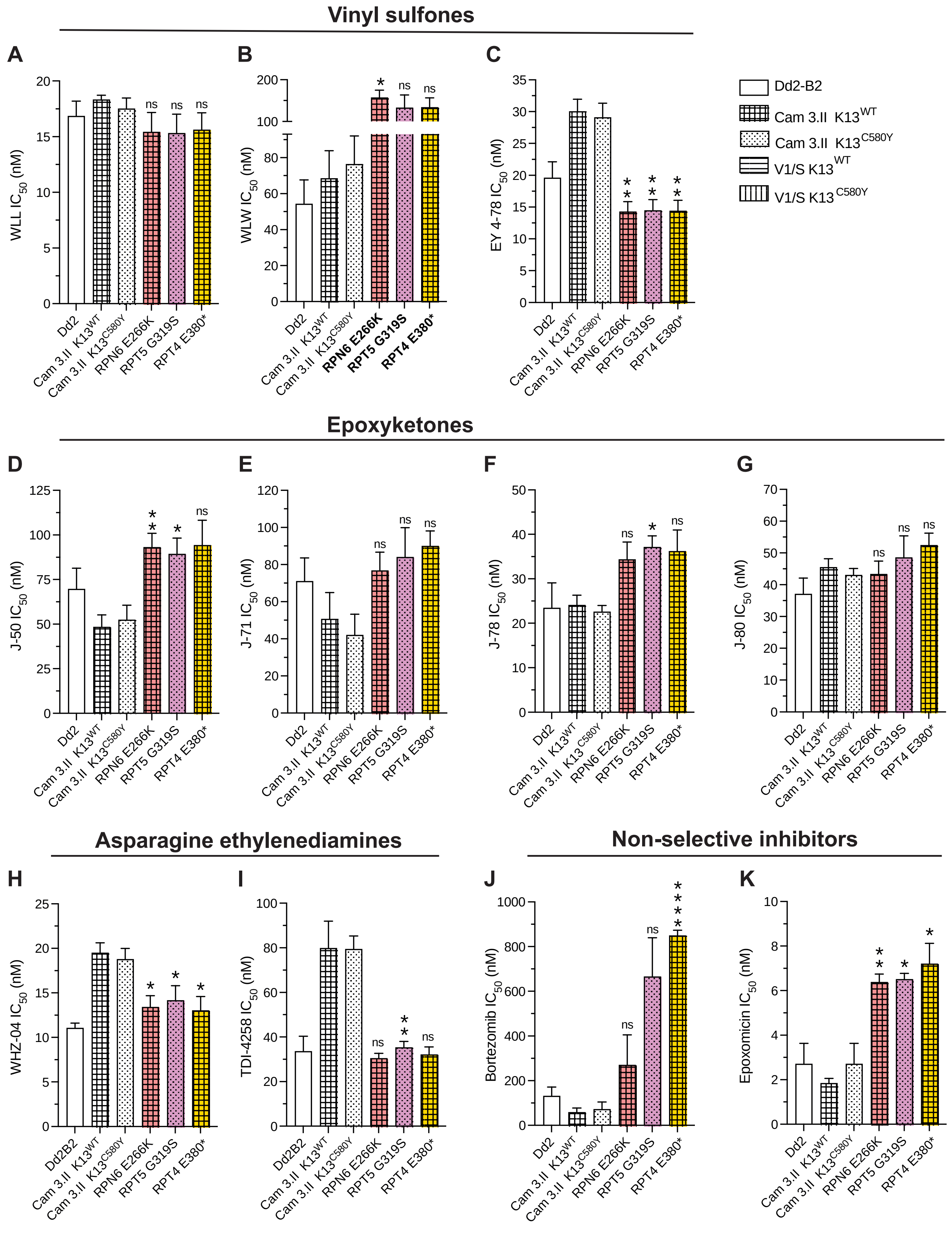

Vinyl sulfones
A
B
C
WLL IC50 (nM)
WLW IC50 (nM)
EY 4-78 IC50 (nM)
Dd2-B2
Cam 3.II K13WT
Cam 3.II K13C580Y
V1/S K13WT
V1/S K13C580Y
Epoxyketones
D
E
F
G
J-50 IC50 (nM)
J-71 IC50 (nM)
J-78 IC50 (nM)
J-80 IC50 (nM)
Asparagine ethylenediamines
Non-selective inhibitors
H
I
J
K
WHZ-04 IC50 (nM)
TDI-4258 IC50 (nM)
Bortezomib IC50 (nM)
Epoxomicin IC50 (nM)
Dd2
Dd2B2
Cam 3.II K13WT
Cam 3.II K13C580Y
RPN6 E266K
RPT5 G319S
RPT4 E380*
ns

Figure S4. Profiling of 19S regulatory particle mutants against *Plasmodium*-selective and non-selective proteasome inhibitors. IC_{50} values for *P. falciparum* 19S proteasome wild-type lines (Dd2, Cam3.II $K13^{WT}$, Cam3.II $K13^{C580Y}$, V1/S $K13^{WT}$ and V1/S $K13^{C580Y}$) and mutant lines tested against *Plasmodium*-selective and non-selective inhibitors in 72 h dose-response assays, as described in **Figure 1**. Compounds tested included: **(A-C)** WLL, WLW and EY 4-78 (vinyl sulfones), **(D-G)** J-50, J-71, J-78 and J-80 (epoxyketones), **(H, I)** WHZ-04 and TDI-4258 (AsnEDAs), **(J)** bortezomib and **(K)** epoxomicin. Lines indicated in bold were obtained from resistance selection studies with the test compound. Results show means ± SEM from 3 to 4 independent assays conducted in duplicate (see **Table S1**). Statistical significance was calculated using unpaired *t* tests with Welch's correction, comparing selected lines to their respective parental lines (detailed in **Table 1** and illustrated here using matched hatching patterns). *$p<0.05$; **$p<0.01$; ***$p<0.001$; ****$p<0.0001$; ns, not significant.

Figure S5

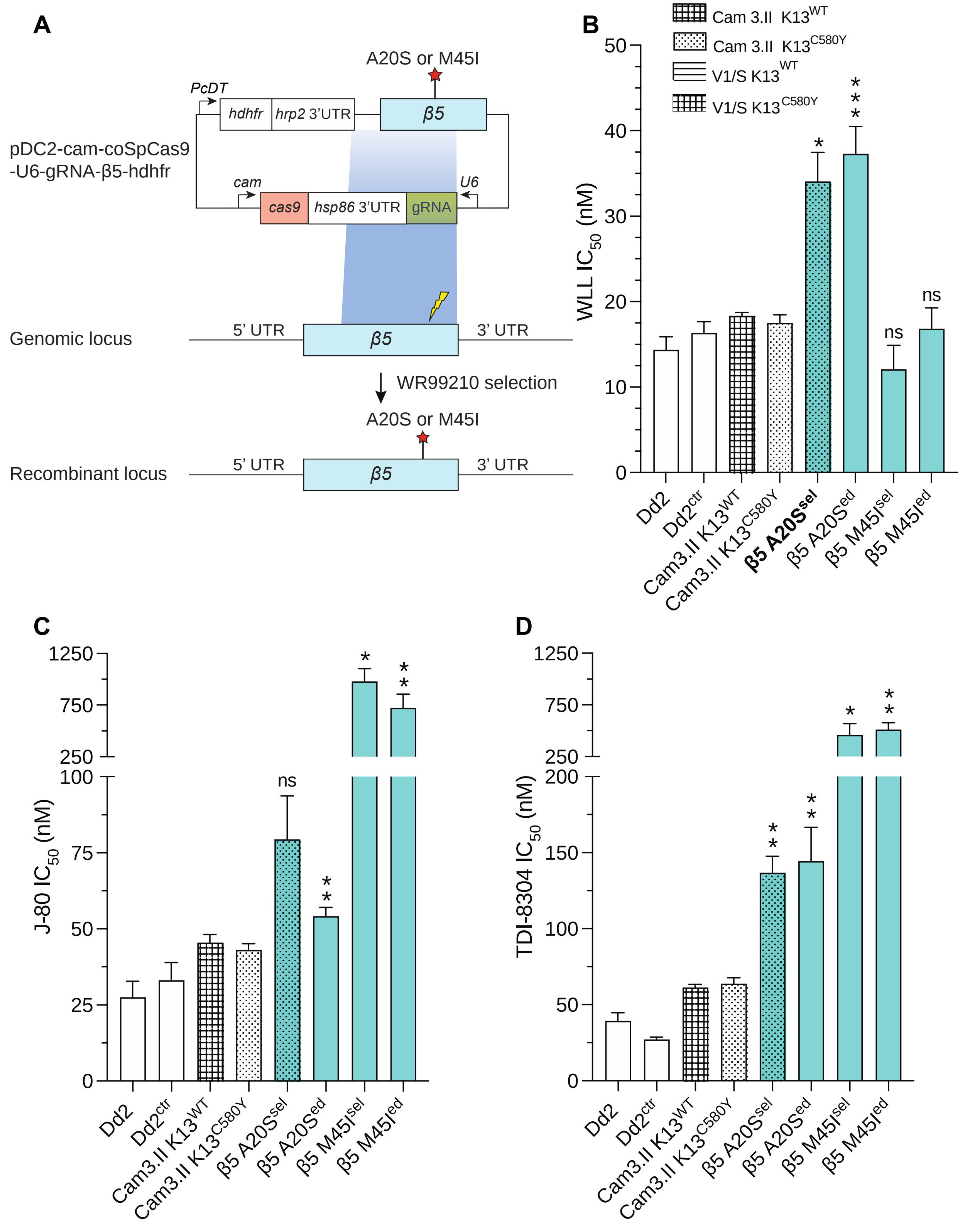
A
A20S or M45I
PcDT
hdhfr
hrp2 3'UTR
β5
pDC2-cam-coSpCas9
-U6-gRNA-β5-hdhfr
cam
U6
cas9
hsp86 3'UTR
gRNA
Genomic locus
5' UTR
3' UTR
WR99210 selection
Recombinant locus
B
Dd2-B2
Cam 3.II K13WT
Cam 3.II K13C580Y
V1/S K13WT
V1/S K13C580Y
WLL IC50 (nM)
ns
Dd2
Dd2ctr
Cam3.II K13WT
Cam3.II K13C580Y
β5 A20Ssel
β5 A20Sed
β5 M45Isel
β5 M45Ied
C
J-80 IC50 (nM)
D
TDI-8304 IC50 (nM)

Figure S5. CRISPR/Cas9-mediated editing of *P. falciparum* 20S β5 proteasome mutations. (A) Schematic of the CRISPR/Cas9 plasmid used to introduce β5 point mutations into wild-type Dd2 parasites. This plasmid includes the *Cas9* and the β5-specific guide RNA (gRNA) expression cassettes (driven by the *calmodulin* and *U6* promoters, respectively), the human dihydrofolate reductase (h*dhfr*) selectable marker (driven by the *P. chabaudi dhfrs-ts* (*Pc*DT) promoter), and a β5-specific template for homology-directed repair. **(B-D)** IC_{50} values for selected or edited β5 A20S, A20V or M45I, and control (ctr) transgenic lines tested against **(B)** WLL, **(C)** J-80, or **(D)** TDI-8304 in 72 h dose-response assays. Bolded lines were selected for resistance to the test compound. Results show means ± SEM from assays conducted on 3 to 5 independent occasions in duplicate (see **Table S4**). Statistical significance was calculated using unpaired *t* tests with Welch's correction, comparing edited lines to the Dd2 parent or selected lines to their respective parental line (detailed in **Table 1** and illustrated here using matched hatching patterns). *p<0.05; **p<0.01; ***p<0.001; ****p<0.0001; ns, not significant.

Figure S6

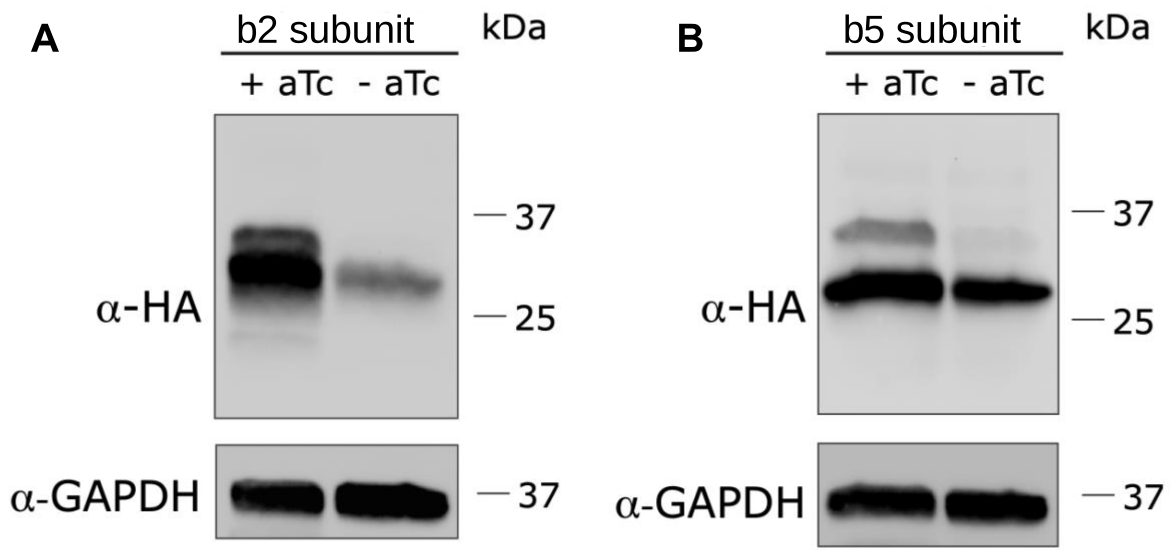

Figure S6. Conditional knock-down parasites demonstrate essentiality for proteasome β2 and β5 subunits. Western blots of **(A)** β2 and **(B)** β5 cKD lines cultured in 500 nM (+aTc) or 0 nM aTc (-aTc). Parasites showed minimal residual protein upon aTc removal. The doublet bands for the subunits represent the precursor and mature forms, present as higher and lower molecular weights. These subunits are initially synthesized as inactive precursors with an N-terminal pro-peptide that is cleaved off to form the mature proteins when the complex is fully assembled[1-3]. Antibodies to GAPDH were used to confirm equivalent protein loading across conditions.

Table S1. IC_{50} values (nM) for inhibitor-selected proteasome mutant lines and proteasome wild-type controls.

	Dd2			Cam 3.II $K13^{WT}$			Cam 3.II $K13^{C580Y}$			V1/S $K13^{WT}$			V1/S $K13^{C580Y}$			β2 C31Y (Cam3.II $K13^{C580Y}$)				β2 C31F (V1/S $K13^{WT}$)				β2 A49E (V1/S $K13^{C580Y}$)			
Compounds	Mean	SEM	N	Mean	SEM	N	Mean	SEM	N	Mean	SEM	N	Mean	SEM	N	Mean	SEM	N	*p* value	Mean	SEM	N	*p* value	Mean	SEM	N	*p* value
**WLL**	16.8	1.3	8	18.3	0.4	3	17.5	1.0	3	11.4	0.8	4	11.1	1.0	4	9.3	1.8	4	0.012	5.8	1.6	4	0.029	15.9	1.6	4	0.047
**WLW**	54.3	13.3	6	68.4	15.4	3	76.4	15.7	3	29.5	3.0	10	25.5	3.2	10	**312**	**55.3**	3	0.042	**245**	**21.8**	4	0.002	**161**	**15.0**	3	0.010
**EY 4-78**	19.6	2.5	4	30.0	1.9	3	29.1	2.2	3	20.9	1.8	4	17.7	1.8	4	5.8	2.1	3	0.002	7.0	1.1	4	0.004	5.9	0.7	4	0.004
**J-50**	69.7	11.7	6	48.5	6.7	3	52.4	8.2	3	24.9	3.2	4	27.0	2.2	4	55.0	2.3	4	0.924	33.4	2.3	4	0.089	55.0	15.1	2	0.310
**J-71**	71.0	12.6	8	50.7	14.3	3	42.1	11.2	3	25.0	2.0	4	25.8	5.8	4	25.9	1.9	3	0.285	47.0	5.6	4	0.024	59.3	9.1	4	0.026
**J-78**	23.4	5.6	5	24.1	2.2	3	22.6	1.4	3	13.9	1.0	4	13.3	0.2	4	12.4	3.1	3	0.065	11.5	2.0	4	0.447	13.4	2.6	3	0.969
**J-80**	37.1	5.0	5	45.5	2.7	3	43.1	2.1	3	27.8	3.2	4	25.5	2.7	4	17.2	4.8	4	0.008	17.9	1.0	4	0.048	27.4	2.8	4	0.636
**WHZ-04**	11.1	0.6	6	19.5	1.1	3	18.8	1.2	3	12.4	1.0	4	11.7	0.7	4	2.7	0.1	4	0.006	3.2	0.2	4	0.002	3.9	0.5	4	0.0002
**TDI-4258**	33.6	6.7	6	79.8	12.1	2	79.4	6.0	2	54.5	3.3	3	50.7	4.5	3	7.1	1.0	4	0.001	7.7	0.6	4	0.004	9.2	0.8	4	0.010
**Compound 4**	269	61.8	2	--	--	--	--	--	--	--	--	--	--	--	--	52.0	25.0	2	0.140	--	--	--	--	--	--	--	--
**Compound 6**	277	28.7	3	--	--	--	--	--	--	--	--	--	--	--	--	81.0	3.3	3	0.020	--	--	--	--	--	--	--	--
**Bortezomib**	132	39.6	8	59.0	18.1	3	72.1	32.4	2	27.9	10.1	4	31.6	9.1	4	168	50.1	4	0.184	204	89.5	4	0.144	231	104	4	0.152
**Epoxomicin**	5.3	1.8	7	1.8	0.2	3	2.7	0.9	3	1.5	0.1	4	1.5	0.1	4	4.0	1.5	4	0.499	4.1	1.0	5	0.065	4.6	1.2	5	0.055

SEM: standard error of the mean; N: number of biological repeats (with technical duplicates); *p* values were determined by comparison between the variant lines and their isogenic proteasome wild-type parental lines using unpaired t tests with Welch's correction. Brackets following mutant parasite names indicate the parental line. Mean and SEM values shown in red with bold text indicate the mutations selected with that particular compound.

Table S1 (continued). IC_{50} values (nM) for inhibitor-selected proteasome mutant lines and proteasome wild-type controls.

	β5 A20S (Cam3.II K13^{C580Y})				β5 A20V (Dd2-B2)				β5 M45I (Dd2-B2)				β5 A49S (Dd2-B2)				β6 A117V (V1/S K13^{WT})				β6 A117D (Dd2-B2)				β6 S208L (V1/S K13^{C580Y})			
Compounds	Mean	SEM	N	p value	Mean	SEM	N	p value	Mean	SEM	N	p value	Mean	SEM	N	p value	Mean	SEM	N	p value	Mean	SEM	N	p value	Mean	SEM	N	p value
**WLL**	**34.1**	**3.4**	3	0.032	7.4	0.8	3	0.000	12.1	2.8	3	0.222	3.7	0.7	3	<0.0001	**34.8**	**2.5**	4	0.001	18.3	2.4	4	0.612	**21.7**	**1.9**	4	0.005
**WLW**	22.7	3.0	3	0.071	6.9	1.9	4	0.016	18.8	0.4	3	0.044	9.0	2.4	4	0.018	165	24.4	4	0.001	10.1	3.8	3	0.020	45.0	3.19	2	0.015
**EY 4-78**	87.1	9.0	4	0.006	47.9	9.4	3	0.087	103	17.6	3	0.040	29.8	4.0	2	0.176	31.5	3.5	4	0.048	65.9	8.1	4	0.008	12.8	1.2	4	0.071
**J-50**	140	24.3	3	0.058	1,402	190	3	0.020	1,160	170	2	0.097	355	113	3	0.127	280	90.0	2	0.216	175	46.5	4	0.106	92.7	9.3	4	0.005
**J-71**	139	16.0	4	0.004	1,004	235	4	0.028	539	186	3	0.128	430	162	3	0.157	142	12.8	4	0.002	187	29.0	4	0.020	70.7	3.6	3	0.002
**J-78**	46.7	7.2	4	0.042	488	179	3	0.122	327	155	2	0.300	174	48.0	3	0.087	56.9	13.2	4	0.018	92.8	24.5	4	0.063	25.3	4.12	4	0.061
**J-80**	79.4	14.3	4	0.083	927	142	4	0.008	979	124	3	0.016	317	132	3	0.167	123	16.5	4	0.009	441	44.6	4	0.003	39.1	3.5	4	0.024
**WHZ-04**	229	6.1	3	0.001	4,619	714	2	0.098	547	120	2	0.141	101	11.9	4	0.005	13.6	1.7	4	0.591	213	26.1	4	0.005	7.4	0.6	4	0.005
**TDI-4258**	525	41.2	4	0.001	>10,000	--	4	--	1344	112	2	0.054	**151**	**19.2**	4	0.005	16.2	1.0	4	0.004	4,200	482	3	0.013	18.9	1.0	4	0.016
**Compound 4**	1,445	269	2	0.129	--	--	--	--	--	--	--	--	--	--	--	--	611	181	2	0.286	--	--	--	--	--	--	--	--
**Compound 6**	1,440	99.0	3	0.004	--	--	--	--	--	--	--	--	--	--	--	--	523	66	3	0.047	--	--	--	--	--	--	--	--
**Bortezomib**	452	49.1	4	0.003	283	108	6	0.234	1,732	4.5	2	<0.0001	178	25.9	4	0.348	1,067	248	3	0.052	980	69.7	3	0.001	535	192	3	0.119
**Epoxomicin**	7.1	2.6	4	0.180	9.5	2.7	5	0.228	6.1	2.2	4	0.798	5.6	1.2	4	0.916	5.9	1.6	5	0.050	5.6	1.7	5	0.897	4.9	1.2	5	0.046

SEM: standard error of the mean; N: number of biological repeats (with technical duplicates); p values were determined by comparison between the variant lines and their isogenic proteasome wild-type parental lines using unpaired t tests with Welch's correction. Brackets following mutant parasite names indicate the parental line. Mean and SEM values shown in red with bold text indicate the mutations selected with that particular compound. Other mutations were selected with compounds not assayed herein: b5 A20V was selected using MMV1579506; b5 M45I was selected using MPI-12; b6 A117D was selected using PKS21004.

Table S2. IC_{50} fold shifts (nM) for inhibitor-selected proteasome mutant lines relative to their respective parental lines.

	β2 C31Y	β2 C31F	β2 A49E	β5 A20S	β5 A20V	β5 M45I	β5 A49S	β6 A117V	β6 A117D	β6 S208L
Compounds	fold shift	fold shift	fold shift	fold shift	fold shift	fold shift	fold shift	fold shift	fold shift	fold shift
**WLL**	0.53	0.51	1.4	**1.9**	0.44	0.72	0.22	**3.1**	1.1	**2.0**
**WLW**	**4.1**	**8.3**	**6.3**	0.30	0.13	0.35	0.17	5.6	0.19	1.8
**EY 4-78**	0.20	0.33	0.33	3.0	2.4	5.2	1.5	1.5	3.4	0.72
**J-50**	1.0	1.3	2.0	2.7	20.1	16.6	5.1	11.2	2.5	3.4
**J-71**	0.62	1.9	2.3	3.3	14.1	7.6	6.1	5.7	2.6	2.7
**J-78**	0.55	0.83	1.0	2.1	20.8	13.9	7.4	4.1	4.0	1.9
**J-80**	0.40	0.64	1.1	1.8	25.0	26.4	8.5	4.4	11.9	1.5
**WHZ-04**	0.14	0.26	0.33	12.2	418	49.5	9.1	1.1	19.2	0.63
**TDI-4258**	0.09	0.14	0.18	6.6	>300	40.0	**4.5**	0.30	125	0.37
**Compound 4**	0.23	-	-	5.2	-	-	-	2.4	-	-
**Compound 6**	0.27	-	-	4.5	-	-	-	1.9	-	-
**Bortezomib**	2.3	7.3	7.3	6.3	2.1	13.1	1.3	38.2	7.4	16.9
**Epoxomicin**	1.5	2.8	3.1	2.6	1.8	1.1	1.0	4.1	1.1	3.3

Parental lines for each mutant are listed in Table 1. Fold shifts were calcaluated using mean values documented in Table S1. Values shown in red with bold text indicate the mutations selected with that particular compound. Other mutations were selected with compounds not assayed herein: b5 A20V was selected using MMV1579506; b5 M45I was selected using MPI-12; b6 A117D was selected using PKS21004.

Table S3. IC_{50} values (nM) for inhibitor-selected proteasome mutant lines and proteasome wild-type controls.

	Dd2			Cam 3.II K13^{WT}			Cam 3.II K13^{C580Y}			RPN6 E266K				RPT5 G319S				RPT4 E380*			
Compounds	Mean	SEM	N	Mean	SEM	N	Mean	SEM	N	Mean	SEM	N	*p* value	Mean	SEM	N	*p* value	Mean	SEM	N	*p* value
**WLL**	16.8	1.3	8	18.3	0.4	3	17.5	1.0	3	15.4	1.7	4	0.193	15.3	1.7	4	0.175	15.6	1.5	4	0.174
**WLW**	54.3	13.3	6	68.4	15.4	3	76.4	15.7	3	**157**	**17.7**	**4**	0.019	**133**	**30.6**	4	0.169	**134**	**22.4**	3	0.111
**EY 4-78**	19.6	2.5	4	30.0	1.9	3	29.1	2.2	3	14.3	1.6	4	0.002	14.5	1.7	4	0.006	14.4	1.6	3	0.004
**J-50**	69.7	11.7	6	48.5	6.7	3	52.4	8.2	3	93.2	7.8	4	0.007	89.3	8.9	4	0.015	94.3	14.0	3	0.063
**J-71**	71.0	12.6	8	50.7	14.3	3	42.1	11.2	3	76.8	9.9	4	0.210	84.0	15.9	4	0.085	90.0	8.1	3	0.092
**J-78**	23.4	5.6	5	24.1	2.2	3	22.6	1.4	3	28.6	6.4	4	0.101	30.0	7.3	4	0.014	36.2	4.8	3	0.111
**J-80**	37.1	5.0	5	45.5	2.7	3	43.1	2.1	3	43.3	4.1	4	0.674	48.6	6.8	4	0.698	52.4	3.8	3	0.220
**WHZ-04**	11.1	0.6	6	19.5	1.1	3	18.8	1.2	3	13.4	1.3	4	0.016	14.2	1.7	4	0.046	13.0	1.6	4	0.020
**TDI-4258**	33.6	6.7	6	79.8	12.1	2	79.4	6.0	2	30.4	2.3	4	0.143	35.3	2.7	4	0.002	32.1	3.4	4	0.1365
**Bortezomib**	132	39.6	8	59.0	18.1	3	72.1	32.4	2	270	134	3	0.276	666	174	3	0.072	850	23.7	3	0.002
**Epoxomicin**	5.3	1.8	7	1.8	0.2	3	2.7	0.9	3	4.5	1.2	5	0.001	5.0	1.0	5	0.045	6.1	1.3	4	0.023

SEM: standard error of the mean; N: number of biological repeats (with technical duplicates); *p* values were determined by comparison between the variant lines and their isogenic proteasome wild-type parental lines using unpaired t tests with Welch's correction. Values shown in red with bold text indicate the mutations selected with that particular compound.

Table S4. IC_{50} values (nM) for CRISPR/Cas9-edited proteasome mutant lines compared with selected lines and proteasome wild-type controls.

	Dd2			Dd2[ctr]				Cam 3.II K13[WT]			Cam 3.II K13[C580Y]			β5 A20S[sel]			
Compound	Mean	SEM	N	Mean	SEM	N	*p* value	Mean	SEM	N	Mean	SEM	N	Mean	SEM	N	*p* value
**WLL**	14.4	1.5	9	16.3	1.3	5	0.506	18.3	0.4	3	17.5	1.0	3	**34.1**	**3.4**	3	0.032
**J-80**	27.5	5.3	5	33.0	5.9	4	0.511	45.5	2.7	3	43.1	2.1	3	79.4	14.3	4	0.083
**TDI-8304**	39.3	5.5	11	27.2	1.5	5	0.055	61.4	2.0	4	63.9	3.9	4	137	10.7	4	0.004

Table S4 (continued). IC_{50} values (nM) for CRISPR/Cas9-edited proteasome mutant lines compared with selected lines and proteasome wild-type controls.

	β5 A20S[ed]				β5 M45I[sel]				β5 M45I[ed]				β5 A20V[sel]				β5 A20V[ed]			
Compound	Mean	SEM	N	*p* value	Mean	SEM	N	*p* value	Mean	SEM	N	*p* value	Mean	SEM	N	*p* value	Mean	SEM	N	*p* value
**WLL**	**37.3**	**3.2**	5	0.001	12.1	2.8	3	0.519	16.8	2.4	5	0.417	7.4	0.8	3	0.003	12.4	2.1	2	0.751
**J-80**	54.1	2.9	5	0.004	979	124	3	0.016	722	134	5	0.007	985	97.2	6	0.0002	1,597	486	3	0.048
**TDI-8304**	144	22.3	5	0.008	460	108	6	0.011	512	66.0	4	0.005	1,140	75.6	3	0.005	1,201	28.8	3	0.0004

SEM, standard error of the mean; N, number of biological repeats (with technical duplicates). β5 A20S[sel]: *P. falciparum* lines generated from selections with WLL. β5 M45I[sel]: *P. falciparum* lines generated from selections with MPI-12. β5 A20S[ed] and β5 M45I[ed]: *P. falciparum* lines generated by introducing β5 A20S and β5 M45I mutations into parental Dd2 (B2 clone) using CRISPR/Cas9. Dd2-B2[ctr]: *P. falciparum* line generated by introducing wild type β5 sequence into parental Dd2-B2 using CRISPR/Cas9. *p* values were determined by comparing the shift in IC_{50} between the variant lines and parental Dd2-B2 using unpaired t tests with Welch's correction. These values were generated as a separate set from those reported in Table S1. Values shown in red with bold text indicate the mutation selected with that particular compound.

Table S5. IC_{50} values (nM) for 20S proteasome β2 and β5 conditional knockdown lines.

	β2 conditional knockdown											β5 conditional knockdown										
	50 nM aTc			20 nM aTc				15 nM aTc				50 nM aTc			20 nM aTc				10 nM aTc			
Compounds	Mean	SEM	N	Mean	SEM	N	*p* value	Mean	SEM	N	*p* value	Mean	SEM	N	Mean	SEM	N	*p* value	Mean	SEM	N	*p* value
**WLL**	7.5	0.6	4	5.6	0.2	4	0.049	3.0	0.4	4	0.002	8.3	0.6	4	7.1	0.5	5	0.146	4.1	0.3	5	0.001
**EY 4-78**	9.3	0.7	4	4.6	0.4	4	0.000	3.2	0.7	4	0.001	7.6	0.2	4	6.3	0.4	4	0.038	4.5	0.2	4	<0.0001
**J-71**	32.0	0.7	4	14.0	1.5	4	0.000	8.3	1.3	4	<0.0001	24.2	1.2	4	23.8	2.1	4	0.873	11.6	1.1	4	0.000
**J-80**	20.1	1.4	4	7.2	0.7	4	0.001	3.5	1.3	4	0.0001	13.1	0.7	4	12.3	0.5	4	0.384	4.8	1.1	4	0.001
**TDI-8304**	24.0	0.6	4	11.9	0.4	4	<0.0001	7.8	0.7	4	<0.0001	10.3	0.4	4	10.1	0.5	4	0.685	5.8	0.3	4	0.0002
**Chloroquine**	2.7	0.2	4	3.0	0.2	4	0.394	3.3	0.25	2	0.199	4.7	0.6	4	4.9	0.4	4	0.789	4.9	0.4	4	0.683

Conditional knockdown lines were generated in NF54 parasites (unlike the selection and gene editing studies performed in other strains and reported in separate tables). SEM: standard error of the mean; N: number of biological repeats (with technical duplicates). *p* values were determined by comparing the IC_{50} values of parasites cultured under 10, 15 or 20 nM aTc (to cause a partial knockdown) with those cultured at 50 nM (no knockdown), using unpaired t tests with Welch's correction.

Table S6. IC_{50} values (nM) for inhibitor-selected proteasome mutant lines and proteasome wild-type control.

	Dd2			β5 M45R				β5 M45V				β5 A50V				β6 N151Y				β6 S157L			
Compounds	Mean	SEM	N	Mean	SEM	N	*p* value	Mean	SEM	N	*p* value	Mean	SEM	N	*p* value	Mean	SEM	N	*p* value	Mean	SEM	N	*p* value
**J-71**	33.8	2.3	16	288	30.0	4	0.003	369	35.0	4	0.002	**104**	**5.5**	4	0.0002	24.1	2.2	4	0.013	71.4	5.7	4	0.003
**J-80**	23.0	1.7	11	**807**	**129**	4	0.004	**266**	**21.7**	4	0.002	69.4	4.4	4	0.001	16.8	2.8	4	0.138	123	9.9	4	0.000
**TDI-8304**	13.3	0.7	9	3.7	0.9	4	<0.0001	8,800	372	3	0.002	25.6	1.0	3	<0.0001	**22.0**	**1.1**	4	0.002	**34,858**	**3,617**	5	0.001
**WLL**	11.9	0.6	11	6.3	0.5	4	<0.0001	6.9	0.5	4	<0.0001	11.7	0.6	4	0.850	8.2	0.8	4	0.008	12.0	1.0	4	0.925
**EY 4-78**	22.1	1.6	11	30.6	3.8	4	0.104	116	11.4	4	0.003	23.3	3.9	4	0.788	11.9	2.3	4	0.011	159	8.0	4	0.0003

SEM: standard error of the mean; N: number of biological repeats (with technical duplicates). β6 S157L and β6 N151Y: *P. falciparum* lines generated from selections with TDI-8304. β5 A50V: *P. falciparum* line generated from selections with J-71. β5 M45V and β5 M45R: *P. falciparum* lines (F2 D9 and F3 A2, respectively) generated from selections with J-80. *p* values were determined by comparing the shift in IC_{50} between the variant lines and parental Dd2-B2 using unpaired t tests with Welch's correction. This data set was generated separately from the data reported in Table S1. Values shown in red with bold text indicate the mutations selected with that particular compound.

Table S7. IC$_{50}$ fold shifts (nM) for selected proteasome mutant lines relative to their parental line Dd2-B2.

	β5 M45R	β5 M45V	β5 A50V	β6 N151Y	β6 S157L
Compounds	fold shift	fold shift	fold shift	fold shift	fold shift
**J-71**	8.5	10.9	**3.1**	0.71	2.1
**J-80**	**35.1**	**11.6**	3.0	0.73	5.3
**TDI-8304**	0.28	662	1.9	**1.7**	**2,621**
**WLL**	0.53	0.58	0.98	0.69	1.0
**EY 4-78**	1.4	5.2	1.1	0.54	7.2

Fold shifts were calcaluated using mean values documented in Table S6. Values shown in red with bold text indicate the mutations selected with that particular compound.

Table S8. Oligonucleotides employed in this study.

Name	Nucleotide sequence (5'-3')	Description	Lab name
p1	TATTATATTAGGAACAATGGCAGG	β5 gRNA 1 BbsI fwd (gRNA for gene editing)	p8349
p2	AAACCCTGCCATTGTTCCTAATAT	β5 gRNA 1 BbsI rev (gRNA for gene editing)	p8350
p3	TATTTAAAAGATCCCATAGATGCT	β5 gRNA 2 BbsI fwd (gRNA for gene editing)	p8351
p4	AAACAGCATCTATGGGATCTTTTA	β5 gRNA 2 BbsI rev (gRNA for gene editing)	p8352
p5	AGAGGTACCGAGCTCGAATTCATGGTAATAGCAAGTGATGAAAGC	β5 gs3226 InFusion EcoRI fwd (donor for gene editing)	p8394
p6	CGAAAAGTGCCACCTGACGTCGAATCTAAAATAGAATAAGCATATGTACTACC	β5 gs3226 InFusion AatII rev (donor for gene editing)	p8395
p7	GTACGGTACAAACCCGGAATTCGAGCTCGGATTTATATCTGTAGAAGATGCATAAGT	β2 RHR fwd (cKD)	NA
p8	GGGTATTAGACCTAGGGATAACAGGGTAATCCTTGAGATATTAAACTACAATAAAAACA	β2 RHR rev (cKD)	NA
p9	TTATATGGGATACATCCCCA	β2 gRNA target site (cKD)	NA
p10	GTACGGTACAAACCCGGAATTCGAGCTCGGACAAAAGGATCAATATGTTATGTGAA	β5 RHR fwd (cKD)	NA
p11	GGGTATTAGACCTAGGGATAACAGGGTAATGGCTAGTCCATAACATTACCT	β5 RHR rev (cKD)	NA
p12	TTTAGAGATGGTGGTTCAGG	β5 gRNA target site (cKD)	NA

cKD, conditional knock-down. Fwd, forward. Rev, reverse. RHR, right homology region. NA, not applicable (cloning performed in the Niles Lab).

Supplementary References

1. Seemuller, E., Lupas, A., and Baumeister, W. (1996). Autocatalytic processing of the 20S proteasome. Nature *382*, 468-471.
2. Chen, P., and Hochstrasser, M. (1996). Autocatalytic subunit processing couples active site formation in the 20S proteasome to completion of assembly. Cell *86*, 961-972.
3. Le Tallec, B., Barrault, M.B., Courbeyrette, R., Guerois, R., Marsolier-Kergoat, M.C., and Peyroche, A. (2007). 20S proteasome assembly is orchestrated by two distinct pairs of chaperones in yeast and in mammals. Mol. Cell *27*, 660-674.
